# The global burden of oesophageal cancer from 1990 to 2023 and projections to 2040: a comprehensive analysis of the Global Burden of Disease Study 2023

**DOI:** 10.7189/jogh.16.04213

**Published:** 2026-07-10

**Authors:** Huajie Xing, Qianjie Xu, Mengyu Hu, Haike Lei, Zhiqiang Wang

**Affiliations:** 1 Department of Thoracic Surgery, Chongqing University Cancer Hospital, Chongqing, China; 2 Chongqing Cancer Multi-omics Big Data Application Engineering Research Center, Chongqing University Cancer Hospital, Chongqing, China; 3 Department of Radiation Oncology, Chongqing University Cancer Hospital, Chongqing, China

## Abstract

**Background:**

Oesophageal cancer (OC) remains a major global public health challenge. This study aims to analyse the global, regional, and national burden of OC from 1990 to 2023 using the latest data from the Global Burden of Disease (GBD) Study 2023.

**Methods:**

Data on EC prevalence, incidence, mortality, and disability-adjusted life years (DALYs) were extracted from GBD 2023. Age-standardised rates (ASRs) were calculated, and temporal trends were assessed using estimated annual percentage changes (EAPCs). Stratified analyses were conducted according to the sociodemographic index (SDI) and risk factor contributions. The disease burden was projected to 2040.

**Results:**

In 2023, there were an estimated 1 075 606 prevalent cases of OC globally, with an ASPR of 11.7 per 100 000 population. Although age-standardised rates declined, the absolute number of cases increased by 67% from 1990 to 2023. The disease burden varied substantially across SDI regions and geographical areas, with sub-Saharan Africa and East Asia bearing the heaviest burden. Tobacco use was the leading risk factor contributing to DALYs. The global ASPR is projected to rise to 18.8 per 100 000 by 2040.

**Conclusions:**

Despite decreases in age-standardised rates, the absolute burden of OC remains high, with significant variations across regions, countries, and SDI levels. Efforts should be taken in prevention and early diagnosis for OC and to better identify high-risk populations to inform targeted prevention and screening, and ultimately reduce the OC burden in an efficient and cost-effective way.

Oesophageal cancer (OC) remains a formidable global public health challenge, characterised by its aggressive nature, poor prognosis, and significant geographical variations in incidence, histology, and aetiology. It ranks as the 11th most commonly diagnosed cancer and the 6th leading cause of cancer-related mortality worldwide [1]. Although evolving dietary practices and social behaviours have contributed to reshaping and improving the global pattern of OC burden, substantial cross-national and cross-regional disparities persist. High OC mortality in regions with low socioeconomic levels continues to impose a significant burden on health care systems and societies [2].

The global pattern of OC burden has undergone considerable changes over the past decade. Significant advancements have been made in screening and treatment, including the adoption of minimally invasive surgery, targeted therapies, immune checkpoint inhibitors, liquid biopsy, and high-quality endoscopic screening, all of which have greatly improved patient outcomes. Reported improvements in 5-year survival rates reflect these advances [3,4]. However, these improvements are largely concentrated in high-income countries with robust health care infrastructure. In many low- and middle-income countries, access to the latest medical technologies remains limited, and survival gains have been modest. In addition, the landscape of OC subtypes is evolving. The two predominant histological subtypes, namely oesophageal squamous cell carcinoma (ESCC) and oesophageal adenocarcinoma (EAC), have distinct etiological drivers and geographical distributions. Oesophageal squamous cell carcinoma remains the most common type globally, accounting for approximately 90% of OC cases. Notably, the incidence of EAC has been rising steadily in western countries, and a similar trend may emerge in the East [5,6]. Furthermore, the COVID-19 pandemic has significantly impacted the diagnosis, management, and outcomes of OC patients. During the pandemic, multidisciplinary care for OC patients was disrupted at multiple stages due to medical resource constraints [7]. These evolving conditions underscore the critical importance of understanding the changing burden of OC to guide effective resource allocation, prioritise research efforts, and implement evidence-based prevention programmes.

The Global Burden of Disease (GBD) study, coordinated by the Institute for Health Metrics and Evaluation (IHME), provides a comprehensive and systematic scientific framework to quantify the magnitude of health loss from major diseases, injuries, and risk factors across ages, sexes, populations, and geographies over time. By analysing GBD data, researchers can track trends in OC incidence, prevalence, mortality, and disability-adjusted life years (DALYs), offering invaluable insights into the effectiveness of current control measures and revealing emerging patterns requiring public health attention [8]. While previous studies have utilised GBD data to describe the burden of OC [9–11], there is a continuous need for updated analyses to monitor recent trends and tailor prevention and control strategies for different regions. This is particularly relevant in the context of evolving risk factor profiles and the implementation of national screening programmes for early detection in high-incidence countries such as China [12]. Furthermore, it's crucial to evaluate the impact of the COVID-19 pandemic on the prevention and treatment of oesophageal cancer, thus providing lessons for the future.

Therefore, this study aims to provide a detailed and up-to-date analysis of the global, regional, and national burden of OC from 1990 to 2023 using the latest data from the GBD study. We will report on prevalence, incidence, mortality, DALYs, and their trends by age, sex, sociodemographic index (SDI), and geographical location.

**Adherence to JoGH’s Guidelines for Reporting Analyses of Big Data Repositories Open to the Public (GRABDROP)**

This study used de-identified, aggregated data from the GBD study. Ethical approval was not required as no individual patient data were involved. This analysis adheres to the JoGH’s Guidelines for Reporting Analyses of Big Data Repositories Open to Public (GRABDROP) [13] and the Guidelines for Accurate and Transparent Health Estimates Reporting (GATHER) recommendations. Completed GRABDROP and GATHER checklists are provided as Table S1 and Table S2 in the **Online Supplementary Document**.

## METHODS

### Data sources and processing

All data used in this study were obtained from the GBD 2023 through the Global Health Data Exchange (GHDx) using the GBD Results Tool (https://vizhub.healthdata.org/gbd-results/). The GBD study, led by the Institute for Health Metrics and Evaluation (IHME), is a comprehensive global epidemiological project that systematically integrates multiple data sources, including population surveillance, disease registries, vital registration systems, and epidemiological surveys. Standardised statistical modelling approaches are applied to generate comparable estimates of disease burden across 204 countries and territories by age, sex, and year.

To ensure reproducibility, data were extracted using a standardised query strategy in the GBD Results Tool. ‘Oesophageal cancer’ was selected as the cause, and data from 1990 to 2023 were retrieved at the global, regional, and national levels. The query parameters were defined as follows: measures included prevalence, incidence, deaths, and DALYs; metrics included numbers and age-standardised rates; locations included global estimates, 21 GBD regions, and 204 countries and territories; age was set to all ages; sex was set to both sexes; and years ranged from 1990 to 2023. All estimates were reported with 95% uncertainty intervals (UIs). No additional data transformation or re-modelling was performed beyond the standard processing conducted by the GBD study.

The estimates reported in the GBD study are not based solely on raw registry data but are generated using a standardised modelling framework developed by the IHME. Specifically, incidence and prevalence estimates were derived using DisMod-MR 2.1, a Bayesian meta-regression tool that synthesises multiple data sources to ensure internal consistency among epidemiological parameters. Mortality estimates were generated using the Cause of Death Ensemble model (CODEm), which integrates diverse data inputs and predictive covariates to produce robust cause-specific mortality estimates. Following the standard GBD analytical framework, DALYs were calculated as the sum of years of life lost (YLLs) and years lived with disability (YLDs). The 95% UIs are presented for all estimates. Rates for prevalence, incidence, DALYs, and mortality are reported per 100 000 population per year. Detailed methodologies employed in the GBD Study have been described previously [14].

The analysis utilised the SDI, a composite measure of sociodemographic development based on income per capita, educational attainment, and total fertility rate. According to the latest GBD classification, regions and countries were categorised into five SDI quintiles: high, high-middle, middle, low-middle, and low SDI.

We analysed the contribution of key risk factors to the OC burden, including tobacco use, alcohol consumption, and dietary risks, as defined in GBD 2023. Population attributable fractions (PAFs) were calculated to estimate the proportion of the burden attributable to each risk factor. The GBD comparative risk assessment framework was used to quantify the proportion of OC burden attributable to each risk factor.

### Data analysis

In this study, the estimated annual percentage change (EAPC) was used to assess temporal trends in age-standardised incidence, mortality, and DALYs rates. The EAPC calculated by fitting a log-linear regression model to the natural logarithm of the age-standardised rates (ASRs) represents the annual change on the logarithmic scale and indicates the random error term. The EAPC and its 95% confidence interval (CI) were derived based on the regression coefficient. Trends were interpreted as increasing or decreasing when the 95% CI excluded 0, and as stable otherwise. Model adequacy was assessed through visual inspection of residual plots to evaluate the assumptions of linearity, homoscedasticity, and normality.

Future trends were projected using a Bayesian Age-Period-Cohort (BAPC) model, which estimates age, period, and cohort effects within a Bayesian framework using Integrated Nested Laplace Approximation (INLA) to generate posterior distributions and 95% credible intervals for projected rates. In the BAPC model, disease incidence was assumed to follow a Poisson distribution, with age, period, and cohort effects modelled using second-order random walk (RW2) priors to ensure smooth temporal transitions. Weakly informative priors were employed to avoid overfitting while allowing the data to primarily guide the posterior estimates. Model convergence and stability were assessed by inspecting the posterior distributions and checking for divergent or unstable estimates. Furthermore, the credibility of the projections was evaluated by comparing recent predicted trends with observed historical patterns to ensure consistency and epidemiological interpretability. These procedures enhance the robustness and reliability of the long-term forecasts.

All statistical analyses and visualisations were conducted using *R*, software (version 4.2.2; R Foundation for Statistical Computing, Vienna, Austria), and a two-sided *P*-value <0.05 was considered statistically significant.

## RESULTS

### Global Burden of OC

**Figure 1:**
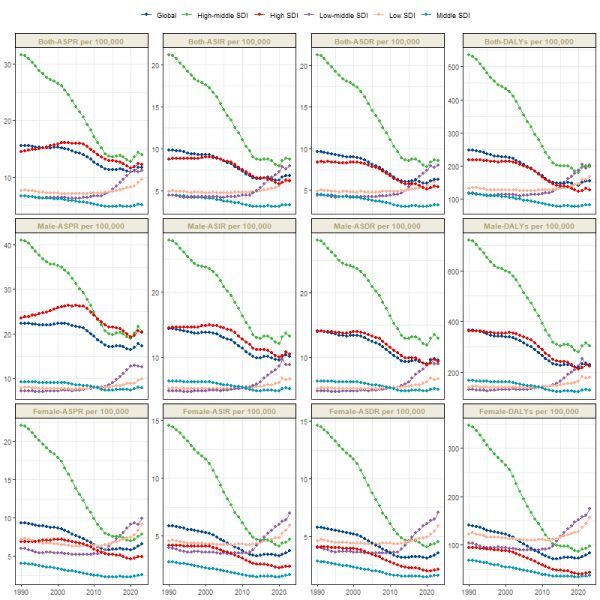
Trends in OC prevalence, incidence, deaths and disability-adjusted life-years from 1990 to 2023. OC – oesophageal cancer.

In 2023, OC remained a significant global health challenge, with an estimated 1 075 606.4 (95% UI = 959 478.3, 1 197 675.8) prevalent cases worldwide, representing a 67% increase since 1990. The ASPR was 11.7 per 100 000 (95% UI = 10.4, 13.0). There were 616 358.8 (95% UI = 551 347.1, 688 595.4) new cases, corresponding to an ASIR of 6.7 per 100 000 (95% UI = 6.0, 7.5), marking a 56% increase from 1990. Oesophageal cancer mortality reached 577 770.3 (95% UI = 505 738.7, 643 198.9) deaths, with an ASDR of 6.3 per 100 000 (95% UI = 5.5, 7.0). The disease resulted in 14 067 301.9 DALYs (95% UI = 12 578 688.2, 15 856 075.8), with an age-standardised DALYs rate of 153 per 100 000 (95% UI = 136.8, 172.8) (**Figure 1**, **Table 1**).

**Table 1:** Global and regional trends in OC burden: prevalence, incidence, mortality, and DALYs (1990–2023)

Location	1990		2023		EAPC 95% CI
	**Number**	**ASR**	**Number**	**ASR**	
**Prevalence**					
Global	642 426.3 (523 170.7, 706 164.1)	15.5 (12.7, 17)	1 075 606.4 (959 478.3, 1 197 675.8)	11.7 (10.4, 13.0)	−1.27 (−1.43, −1.11)
High SDI	317 146.6 (276 487.5, 349 482.4)	14.5 (12.6, 16)	523 066.6 (470 198.1, 576 783)	12.2 (11.0, 13.5)	−0.82 (−1.05, −0.59)
High-middle SDI	240 372.6 (154 078.2, 276 923.6)	31.6 (20.5, 36.4)	291 062.1 (254 544.3, 320 241.1)	14.0 (12.2, 15.4)	−3.15 (−3.41, −2.89)
Middle SDI	23 810 (20 665.7, 27 389.2)	6.6 (5.8, 7.7)	48 031.5 (40 594.1, 60 157.2)	5.1 (4.3, 6.3)	−1.13 (−1.26, −1)
Low-middle SDI	28 853.8 (23 017.7, 35 850.5)	6.6 (5.4, 8.3)	112 943.9 (84 120.2, 147 913.3)	11.2 (8.4, 14.6)	1.62 (1.2, 2.05)
Low SDI	31 990.9 (23 819.5, 42 260.1)	7.5 (5.7, 10)	100 073.2 (73 402, 140 255.8)	9.5 (7.0, 13.2)	0.39 (0.18, 0.6)
Andean Latin America	584.8 (501.7, 669.4)	2.7 (2.4, 3.1)	1150.9 (966.7, 1391.5)	1.8 (1.5, 2.1)	−1.49 (−1.64, −1.34)
Australasia	2244.7 (1999.2, 2534.5)	9.6 (8.5, 10.8)	6429.4 (5287.8, 7838.9)	11.8 (9.9, 14.4)	0.61 (0.35, 0.86)
Caribbean	1682.9 (1569, 1805.2)	6.3 (5.8, 6.7)	3379.1 (3001.2, 3808.8)	6.0 (5.3, 6.8)	0.2 (0.04, 0.37)
Central Asia	9671.9 (9087.7, 10151.7)	20.2 (19, 21.2)	6053.2 (5664.5, 6551.9)	6.4 (6.0, 6.9)	−3.62 (−3.83, −3.41)
Central Europe	7096.3 (6742, 7459)	4.7 (4.5, 4.9)	8843.7 (8240.3, 9386.7)	4.3 (4.0, 4.6)	−0.42 (−0.58, −0.26)
Central Latin America	3416.4 (3099, 3712.9)	3.9 (3.6, 4.3)	6140.3 (5566.3, 6757.2)	2.3 (2.0, 2.5)	−1.61 (−1.67, −1.54)
Central sub-Saharan Africa	2626.7 (1449.1, 3705.5)	10.1 (5.6, 14.2)	8185 (5209.9, 12 092.5)	11.6 (7.4, 17.2)	0.27 (0.08, 0.46)
East Asia	386 329.1 (265 147.5, 44 6928.7)	41 (28.5, 47.4)	466 275.5 (407 689.6, 523 369.0)	19.4 (17.0, 21.7)	−2.92 (−3.21, −2.62)
Eastern Europe	19 081.3 (17 339.2, 20 887)	6.6 (6, 7.3)	17 896.4 (16 196.1, 19 771.8)	5.1 (4.6, 5.7)	−0.85 (−1.04, −0.66)
Eastern sub-Saharan Africa	14 839.4 (10 476.5, 19 991.6)	16.4 (11.7, 22)	40 208.7 (30 202.4, 54 679.3)	17.6 (13.3, 24.0)	0.17 (0.02, 0.32)
High-income Asia Pacific	32 300.5 (28 871.9, 36 123.5)	15.7 (14, 17.6)	77 582.1 (68 427.5, 85 315.6)	17.4 (15.7, 19.2)	0.12 (−0.23, 0.46)
High-income North America	28 419.9 (24 965.6, 32 697.8)	8.5 (7.5, 9.8)	56 119.9 (48 435.8, 63 589.7)	9.0 (7.8, 10.2)	−0.02 (−0.22, 0.18)
North Africa and Middle East	6560.3 (4903.2, 83 18.2)	3.6 (2.7, 4.6)	16 525.7 (13 400.8, 21 207.7)	3.2 (2.6, 4.0)	−0.73 (−0.84, −0.61)
Oceania	130.7 (85.5, 184.1)	4.0 (2.6, 5.6)	360.1 (249.3, 533.5)	3.9 (2.7, 5.7)	−0.28 (−0.35, −0.21)
South Asia	41 714.6 (32 915.6, 53 565.7)	6.5 (5.1, 8.4)	194 636.2 (151 837.8, 250 940.8)	11.7 (9.1, 15.0)	1.67 (1.24, 2.10)
Southeast Asia	13 106.6 (10 235.5, 16 779)	4.6 (3.6, 5.8)	38 591.1 (31 578.5, 48 664.1)	5.0 (4.1, 6.3)	0.10 (0.02, 0.17)
Southern Latin America	5263 (4961.3, 5622.9)	11.3 (10.6, 12)	5710.5 (5126.1, 6303.9)	6.2 (5.6, 6.9)	−2.02 (−2.20, −1.84)
Southern sub-Saharan Africa	6544.7 (5033.8, 8392.9)	22.2 (17.1, 28.4)	11 105.1 (9341.6, 13 188.1)	15.3 (13.0, 18.1)	−1.65 (−1.92, −1.39)
Tropical Latin America	10 089.7 (9047.2, 11 025.2)	10.2 (9.2, 11.1)	19 778.2 (18 408.7, 21 059.9)	7.2 (6.7, 7.7)	−0.87 (−1.00, −0.74)
Western Europe	48 095.2 (44 764.9, 52 135.7)	9.0 (8.4, 9.7)	83 535.9 (71 926, 96 477.2)	9.9 (8.6, 11.4)	0.25 (0.13, 0.37)
Western sub-Saharan Africa	2627.6 (1926.3, 3464.5)	2.7 (2.0, 3.6)	7099.3 (5095.6, 10 131.4)	2.9 (2.1, 4.1)	0.11 (−0.04, 0.26)
**Incidence**					
Global	395 048.6 (330 522.6, 435 204.5)	9.8 (8.3, 10.8)	616 358.8 (551 347.1, 688 595.4)	6.7 (6.0, 7.5)	−1.62 (−1.8, −1.44)
High SDI	190 890.2 (166 983.3, 209 893.3)	8.8 (7.7, 9.6)	271 886.9 (245 915.2, 296 489.7)	6.1 (5.6, 6.7)	−1.45 (−1.66, −1.24)
High-middle SDI	152 426.2 (102 467.2178 402.3)	21.2 (14.4, 24.8)	178 928.6 (155 370.7, 198 624.1)	8.7 (7.6, 9.7)	−3.39 (−3.65, −3.12)
Middle SDI	14 996.5 (12 862.6, 17 351)	4.4 (3.8, 5.2)	29 254.2 (24 953.7, 36 157.1)	3.3 (2.8, 4.0)	−1.29 (−1.44, −1.15)
Low-middle SDI	17 746.7 (13 640, 21 753)	4.4 (3.4, 5.4)	74 719.5 (55 719.6, 97 081.2)	7.9 (5.9, 10.2)	1.76 (1.31, 2.21)
Low SDI	18 829.4 (13 908, 24 233.3)	4.8 (3.6, 6.3)	61 311.4 (44 519.2, 87 453)	6.3 (4.6, 9.0)	0.52 (0.32, 0.71)
Andean Latin America	389.9 (336.4, 441.4)	2.0 (1.7, 2.2)	927.9 (780.5, 1121.8)	1.4 (1.2, 1.7)	−1.43 (−1.64, −1.21)
Australasia	1175.3 (1083.7, 1260.7)	5.0 (4.6, 5.4)	2713.4 (2379.5, 3058.1)	4.7 (4.2, 5.3)	−0.27 (−0.45, −0.09)
Caribbean	1087 (1019.2, 1149.7)	4.1 (3.8, 4.3)	2141.3 (1909.3, 2370.8)	3.8 (3.4, 4.2)	0.03 (−0.15, 0.21)
Central Asia	6277.6 (5922, 6573.3)	13.8 (13.0, 14.4)	3836.4 (3589.2, 4132)	4.3 (4.0, 4.6)	−3.66 (−3.85, −3.47)
Central Europe	4598.6 (4354.6, 4833.8)	3.1 (2.9, 3.2)	5645.7 (5307.1, 5958.2)	2.6 (2.5, 2.8)	−0.6 (−0.73, −0.47)
Central Latin America	2258.1 (2067.5, 2442.9)	2.8 (2.5, 3.0)	3956.9 (3614.3, 4305.5)	1.5 (1.4, 1.6)	−1.87 (−1.95, −1.79)
Central sub-Saharan Africa	1618.5 (965.5, 2249.7)	7.0 (4.2, 9.7)	5302.6 (3534.6, 7722)	8.4 (5.6, 12.4)	0.34 (0.16, 0.51)
East Asia	246 318.8 (175 140.2, 289 116.1)	27.6 (19.9, 32.3)	275 283.6 (239 158.4, 314 351.6)	11.4 (9.8, 13.0)	−3.35 (−3.66, −3.04)
Eastern Europe	12 277.8 (11 170.4, 13 335.3)	4.3 (3.9, 4.7)	10 678.9 (9715.6, 11 698.1)	3.0 (2.7, 3.2)	−1.22 (−1.37, −1.06)
Eastern sub-Saharan Africa	8513.5 (6186.1, 11 130.8)	10.2 (7.5, 13.3)	24 854.9 (18 967.7, 33 998.3)	11.9 (9.0, 16.1)	0.34 (0.22, 0.47)
High-income Asia Pacific	13 699 (12 652.1, 14 616.8)	6.8 (6.2, 7.2)	26 591 (23 714.1, 28 839.9)	5.5 (4.9, 5.9)	−0.86 (−1.05, −0.68)
High-income North America	14812 (13 502.4, 16 124.1)	4.3 (4.0, 4.7)	27 749.2 (24 819.4, 30 316.2)	4.2 (3.8, 4.6)	−0.21 (−0.37, −0.06)
North Africa and Middle East	4127.8 (3154.6, 5203.1)	2.5 (1.9, 3.2)	9422.2 (7665, 12 129.3)	2.0 (1.6, 2.6)	−0.96 (−1.08, −0.84)
Oceania	79.6 (52.3, 111.9)	2.8 (1.8, 3.8)	218.6 (151.2, 327.3)	2.7 (1.9, 4.0)	−0.29 (−0.36, −0.22)
South Asia	25 511.9 (19 497.4, 33 089.2)	4.3 (3.3, 5.6)	124 870.4 (96 956.7, 159 930.1)	8 (6.2, 10.3)	1.78 (1.34, 2.23)
Southeast Asia	7795.4 (6129.3, 9725.6)	2.9 (2.3, 3.7)	21 797.3 (17 420.1, 27 137.7)	2.9 (2.3, 3.6)	−0.18 (−0.26, −0.1)
Southern Latin America	3592.8 (3407.1, 3801.8)	7.8 (7.4, 8.3)	3757.4 (3376.6, 4087.7)	4.0 (3.6, 4.3)	−2.25 (−2.43, −2.07)
Southern sub-Saharan Africa	3900 (2950, 4920.2)	13.9 (10.6, 17.5)	6431.3 (5454.9, 7561.5)	9.4 (8.0, 11)	−1.74 (−1.97, −1.51)
Tropical Latin America	6286.8 (5651.8, 6831.3)	6.8 (6.1, 7.3)	13 074.1 (12 172.7, 13 886.9)	4.8 (4.5, 5.1)	−1.00 (−1.1, −0.89)
Western Europe	29 073.1 (27 470.2, 30 530.7)	5.2 (4.9, 5.4)	42 490.2 (38 560.7, 45 989.3)	4.6 (4.2, 5.0)	−0.52 (−0.59, −0.45)
Western sub-Saharan Africa	1655.1 (1221.7, 2160.3)	1.8 (1.4, 2.4)	4615.6 (3284.8, 6370.3)	2.1 (1.5, 2.8)	0.27 (0.14, 0.39)
**Deaths**					
Global	381 477.4 (321 427.4, 422 498.4)	9.6 (8.2, 10.6)	577 770.3 (505 738.7, 643 198.9)	6.3 (5.5, 7.0)	−1.77 (−1.95, −1.58)
High SDI	182 779.3 (160 144.7, 201 655.1)	8.4 (7.4, 9.3)	244 352.2 (221 022.6, 266 003.8)	5.4 (5.0, 5.9)	−1.73 (−1.94, −1.52)
High-middle SDI	148 506.8 (101 014.7, 174 222.6)	21.2 (14.7, 24.8)	173 158.5 (150 611.4, 191 167.4)	8.5 (7.4, 9.4)	−3.45 (−3.72, −3.19)
Middle SDI	14 662.1 (12 558.3, 17 043.4)	4.5 (3.9, 5.3)	28 141.4 (24 067.5, 34 562.2)	3.2 (2.8, 4.0)	−1.36 (−1.5, −1.21)
Low-middle SDI	17 207.8 (13 282, 21 103.1)	4.5 (3.5, 5.5)	73 405.8 (54 547.3, 95 371.5)	8.0 (5.9, 10.4)	1.78 (1.32, 2.24)
Low SDI	18 164 (13 448, 23 378.1)	4.9 (3.6, 6.3)	58 757.9 (42 644.6, 83 683.5)	6.3 (4.6, 9.0)	0.5 (0.32, 0.68)
Andean Latin America	404.4 (349.3, 458.5)	2.1 (1.8, 2.4)	961.6 (812.0, 1162.1)	1.5 (1.3, 1.8)	−1.5 (−1.71, −1.3)
Australasia	1040.8 (964.5, 1113.4)	4.4 (4.1, 4.7)	2150.6 (1934.0, 2355.0)	3.6 (3.2, 3.9)	−0.74 (−0.89, −0.6)
Caribbean	1097.6 (1033.1, 1160.2)	4.2 (3.9, 4.4)	2097.8 (1883.8, 2322.6)	3.7 (3.3, 4.1)	−0.1 (−0.29, 0.08)
Central Asia	6246.6 (5887.2, 6537)	14.1 (13.3, 14.8)	3788.8 (3547.7, 4107.7)	4.4 (4.1, 4.7)	−3.67 (−3.85, −3.48)
Central Europe	4559.2 (4318.2, 4791.3)	3.1 (2.9, 3.2)	5617.7 (5293.2, 5906.1)	2.6 (2.4, 2.7)	−0.67 (−0.79, −0.56)
Central Latin America	2305.4 (2111.9, 2492.4)	2.9 (2.7, 3.2)	4003.8 (3665.9, 4346.3)	1.5 (1.4, 1.7)	−1.98 (−2.06, −1.9)
Central sub-Saharan Africa	1561.5 (934.9, 2164.4)	7.2 (4.3, 10.0)	5144.9 (3429.9, 7540.5)	8.7 (5.7, 12.9)	0.33 (0.16, 0.49)
East Asia	240 667.2 (173 058.6, 282 288.2)	27.8 (20.4, 32.5)	261 368 (223 690.6, 299 941.4)	10.9 (9.3, 12.4)	−3.52 (−3.84, −3.19)
Eastern Europe	12 044.6 (10 952.3, 13 017.3)	4.2 (3.9, 4.6)	10 119.2 (9201.1, 11 016.6)	2.8 (2.5, 3.0)	−1.38 (−1.53, −1.24)
Eastern sub-Saharan Africa	8108.2 (5921.1, 10 580.4)	10.2 (7.5, 13.3)	23 658.2 (18 116.1, 32 278.4)	11.8 (9.0, 16.0)	0.34 (0.22, 0.45)
High-income Asia Pacific	10 683.8 (10 016.6, 11 256.7)	5.3 (5.0, 5.6)	17 852.7 (15 767.5, 19 387.1)	3.5 (3.1, 3.7)	−1.56 (−1.65, −1.47)
High-income North America	13 101.4 (12 104.4, 14 030.5)	3.8 (3.5, 4.0)	24 100 (22 010.5, 26 196.7)	3.5 (3.2, 3.8)	−0.28 (−0.41, −0.16)
North Africa and Middle East	4049.6 (3072.8, 5109.7)	2.6 (2.0, 3.3)	8919.8 (7228.8, 11 447.8)	2.0 (1.6, 2.6)	−1.03 (−1.15, −0.91)
Oceania	77 (50.8, 107.4)	2.9 (1.9, 3.9)	208.1 (144.9, 310.7)	2.8 (2.0, 4.1)	−0.32 (−0.39, −0.24)
South Asia	24 682 (18 965.3, 31 913)	4.4 (3.3, 5.6)	122 202.2 (94 809.1, 156 867.1)	8.1 (6.3, 10.4)	1.8 (1.35, 2.25)
Southeast Asia	7483.5 (5856.1, 9377.9)	2.9 (2.3, 3.7)	20 217.6 (15 957.5, 25 129.1)	2.8 (2.2, 3.4)	−0.34 (−0.42, −0.27)
Southern Latin America	3683.6 (3496.7, 3895.4)	8.2 (7.7, 8.6)	3852.1 (3473.2, 4205.1)	4.0 (3.6, 4.4)	−2.34 (−2.52, −2.16)
Southern sub-Saharan Africa	3822.5 (2912.1, 4827.7)	13.9 (10.6, 17.6)	6329.4 (5410.8, 7452.1)	9.5 (8.1, 11.2)	−1.71 (−1.94, −1.49)
Tropical Latin America	6150.2 (5531, 6680.7)	6.8 (6.2, 7.4)	12 869.7 (12 005.1, 13 676.8)	4.7 (4.4, 5.0)	−1.06 (−1.16, −0.96)
Western Europe	28 092.7 (26 583.8, 29 357.1)	4.9 (4.7, 5.1)	37 847.2 (34 366.7, 40 246.0)	3.9 (3.6, 4.1)	−0.91 (−0.97, −0.85)
Western sub-Saharan Africa	1615.6 (1197.4, 2101.2)	1.8 (1.4, 2.4)	4460.9 (3175.5, 6137.1)	2.1 (1.5, 2.8)	0.26 (0.14, 0.38)
**DALYs**					
Global	10 267 455.8 (8 534 468.4, 11 344 090)	247.6 (206.7, 273.5)	14 067 301.9 (12 578 688.2, 15 856 075.8)	153.0 (136.8, 172.8)	−1.98 (−2.18, −1.78)
High SDI	4 743 339.2 (4 116 379.4, 5 242 317.3)	217.3 (188.4, 239.8)	5 468 565.9 (5 020 626.6, 5 965 745.1)	127.9 (118.0, 138.8)	−2.03 (−2.26, −1.8)
High-middle SDI	4 041 789.2 (2 708 679.1, 4 666 913.3)	534.8 (362.0, 621.9)	4 076 546.9 (3 615 771.1, 4 496 003.6)	196.1 (174.1, 216.3)	−3.76 (−4.04, −3.48)
Middle SDI	415 102.5 (354 890.3, 476 050.1)	115.5 (98.8, 133.8)	759 467.6 (648 800.7, 935 897.2)	80.4 (68.5, 99.3)	−1.44 (−1.58. −1.3)
Low-middle SDI	507 408.5 (387 574.2, 623 088.4)	117.1 (90.2, 143.6)	2 015 237.5 (1 505 880.1, 2 621 968.6)	201.4 (150.1, 261.9)	1.64 (1.2, 2.07)
Low SDI	555 621.3 (406 511.1, 718 741.2)	131.7 (97.1, 169.4)	1 747 847.2 (1 273 740.5, 2 494 113.8)	167.3 (121.4-238.6)	0.44 (0.24, 0.65)
Andean Latin America	9935.8 (8584.4, 11 249.6)	46.8 (40.4, 53.0)	21 622.3 (18 276.7, 25 797.6)	33.1 (27.9, 39.5)	−1.53 (−1.76, −1.31)
Australasia	23 465.1 (21 884, 25 111.8)	100.6 (93.8, 107.6)	42 653.5 (38 720.5, 46 389.3)	78.5 (71.5, 85.2)	−0.84 (−0.98, −0.69)
Caribbean	27 342.5 (25 619.5, 28 991.2)	101.8 (95.5, 107.8)	53 790.2 (48 236, 59 793.3)	95.5 (85.4, 106.2)	0.09 (−0.1, 0.27)
Central Asia	168 012.4 (158 181.6, 175 942.4)	351.2 (330.5, 367.8)	100 458.1 (94 259.8, 108 740.9)	106.6 (99.9, 115.0)	−3.79 (−3.98, −3.6)
Central Europe	124 932.9 (118 635.3, 131 285.1)	83.0 (78.9, 87.2)	137 682.6 (129 744.9, 145 133.6)	67.3 (63.4, 71.0)	−0.8 (−0.95, −0.65)
Central Latin America	57 549.1 (52 958.3, 62 193.8)	66.6 (61.2, 72.0)	94 340.9 (86 243.5, 102 197.3)	34.7 (31.7, 37.5)	−1.9 (−1.99, −1.81)
Central sub-Saharan Africa	47 460 (28 147.2, 65 715.8)	183.9 (109.7, 254.9)	151 466.2 (102 393.6, 219 053.3)	217 (144.9, 317.5)	0.29 (0.1, 0.48)
East Asia	6 482 794.1 (4 575 836.6, 7 572 522.2)	692.7 (494.4, 811.5)	5 920 498 (5 269 283.4, 6 748 331.3)	244.4 (217.2, 277.6)	−3.87 (−4.21, −3.54)
Eastern Europe	326 678.2 (297 203.2, 355 673.3)	113.7 (103.6, 123.8)	268 225.4 (244 818.8, 294 602.9)	76.5 (69.8, 83.8)	−1.34 (−1.49, −1.19)
Eastern sub-Saharan Africa	258 199 (185 468.8, 34 0376.2)	287.6 (208.2, 375.8)	735 805 (564 345.6, 1 011 702.1)	324.2 (247.5, 443.9)	0.26 (0.13, 0.39)
High-income Asia Pacific	267 546.3 (252 303.4, 281 926.5)	129.6 (122, 136.6)	328 144.3 (298 251.9, 354 184)	74.4 (68.7, 79.7)	−2.00 (-2.13, −1.88)
High-income North America	316 810.6 (294 945.9, 337 699)	95.9 (89.2, 102.2)	533 290.8 (488 703.1, 575 984)	84.3 (77.2, 91.2)	−0.48 (−0.6, −0.36)
North Africa and Middle East	109 473.9 (83 078.2, 137 720.7)	61.7 (46.8, 77.7)	226 381.1 (181 879.8, 292 242.9)	44.5 (36.0, 57.3)	−1.28 (−1.4, −1.16)
Oceania	2241.4 (1463.4, 3148.8)	69.5 (45.9, 96.7)	6120.1 (4239.3, 9235.3)	67.6 (47.4, 100.9)	−0.3 (−0.38, −0.23)
South Asia	737 978.5 (561 208.6, 955 034.3)	114.6 (87.6, 148.6)	3 357 366.6 (2 617 990.4, 4 288 885.9)	204.0 (159.2, 261.1)	1.63 (1.2, 2.07)
Southeast Asia	221 959 (173 196.8, 278 854.9)	77.9 (60.9, 97.5)	589 200.2 (468 354.8, 739 397.2)	76.0 (60.3, 95.0)	−0.24 (−0.31, −0.16)
Southern Latin America	86 682.1 (82 569.4, 91 481.2)	186.3 (177.5, 196.5)	81 013.7 (73 502.3, 88 179.2)	87.6 (79.6, 95.4)	−2.46 (−2.63, −2.28)
Southern sub-Saharan Africa	108 766 (81 777.9, 137 863.3)	370.2 (279.5, 466.8)	175 436.4 (147 245.9, 207 640.1)	244.7 (206.9, 288.2)	−1.82 (−2.05, −1.59)
Tropical Latin America	173 346.2 (156 163, 188 310.2)	176 (158.8, 191)	334 044.9 (312 188.3, 353 950.8)	121.8 (113.9, 128.9)	−1.08 (−1.2, −0.96)
Western Europe	668 942.2 (641 226.6, 696 279)	124.2 (119.3, 129.2)	777 571.7 (719 210.8, 823 804.3)	90.5 (84.1, 96.0)	−1.17 (−1.24, −1.1)
Western sub-Saharan Africa	47 340.4 (34 575.3, 62 277.6)	49.3 (36.2, 64.6)	132 189.9 (94 196.4, 182 735.4)	54.3 (38.7, 74.9)	0.21 (0.07, 0.35)

ASR – age-standardised rates, CI – confidence interval, DALYs – disability-adjusted life years, EAPC – estimated annual percentage change, OC – oesophageal cancer, SDI – sociodemographic index

From 1990 to 2023, the global ASPR of OC declined significantly, with an EAPC of −1.27 (95% CI = −1.43, −1.11). The ASIR also decreased (EAPC = −1.62; 95% CI: −1.80, −1.44), while the ASDR and age-standardised DALYs rate showed EAPCs of −1.77 (95% CI = −1.95, −1.58) and −1.98 (95% CI = −2.18, −1.78), respectively. These trends indicate consistent improvements in disease management and prevention over the past three decades (**Figure 1**, **Table 1**).

### SDI and regional variations

The burden of OC varied substantially across SDI quintiles. High-middle SDI regions had the highest ASPR at 14.0 per 100 000 (95% UI = 12.2, 15.4), while middle SDI regions reported the lowest at 5.1 per 100 000 (95% UI = 4.3, 6.3) (**Figure 1**, **Table 1**). Temporal trends in ASPR showed varied patterns across SDI levels, reflecting different stages of epidemiological transition. The most significant increase in ASPR was observed in low-middle SDI regions (EAPC = 1.62; 95% CI = 1.20, 2.05), indicating a growing burden in these areas. In contrast, high-middle SDI regions exhibited the most substantial and consistent declines across all ASRs (ASPR EAPC = −3.15; ASIR EAPC = −3.39; ASDR EAPC = −3.45; DALYs rate EAPC = −3.76) (**Figure 1**, **Table 1**). In these regions, the ASPR decreased from 31.6 per 100 000 in 1990 to 14.0 per 100 000 in 2023.

**Figure 2:**
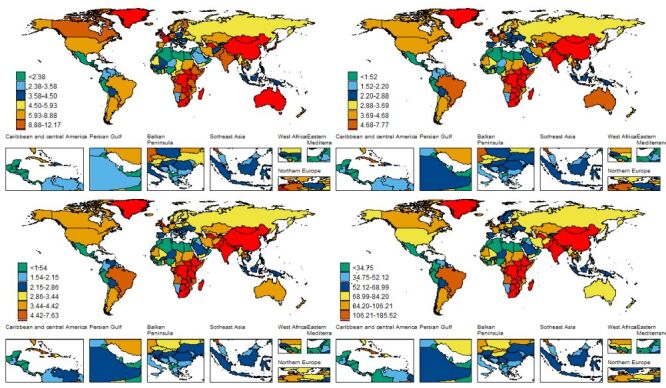
The global disease burden of OC for both sexes in 204 countries and territories. **Panel A.** Prevalence rate. **Panel B.** Incidence rate. **Panel C.** Death rate. **Panel D.** DALYs rate. DALYs – disability-adjusted life years, OC – oesophageal cancer.

Significant regional disparities were also observed. East Asia bore the highest ASPR (19.4 per 100 000), followed by Eastern sub-Saharan Africa (17.6 per 100 000). The lowest ASPR was observed in Andean Latin America (1.8 per 100 000). Eastern sub-Saharan Africa reported the highest ASIR (11.9 per 100 000) and ASDR (11.8 per 100 000), followed closely by East Asia (ASIR = 11.4 per 100 000; ASDR = 10.9 per 100 000). Eastern sub-Saharan Africa also had the highest DALYs rate (324.2 per 100 000). This concentration of high disease burden highlights differences not only in risk factors exposure but also in genetic predisposition, quality of cancer care, and secondary prevention efforts. Andean and Central Latin America were among the regions with the lowest ASRs (**Figure 2**, **Table 1**). These findings collectively underscore the complex relationship between sociodemographic factors, regional disparities, and OC outcomes.

Notably, temporal trends from 1990 to 2023 showed that South Asia experienced the most significant increases in all ASRs (ASPR EAPC = 1.67; 95% CI = 1.24, 2.1); ASIR EAPC = 1.78 (95% CI = 1.34, 2.23); ASDR EAPC = 1.8 (95% CI = 1.35, 2.25); DALYs rate EAPC = 1.63 (95% CI = 1.2, 2.07)). Conversely, Central Asia experienced the most significant declines in ASPR, ASIR, and ASDR (ASPR EAPC = −3.62 (95% CI = −3.83, −3.41); ASIR EAPC = −3.66 (95% CI = −3.85, −3.47); ASDR EAPC = −3.67 (95% CI = −3.85, −3.48)), while the steepest decline in the DALYs rate was observed in East Asia (EAPC = −3.87 (95% CI = −4.21, −3.54)) (**Table 1**). These contrasting trends within Asia underscore the complex and varying dynamics of OC across regions, highlighting the need for targeted interventions.

**Figure 3:**
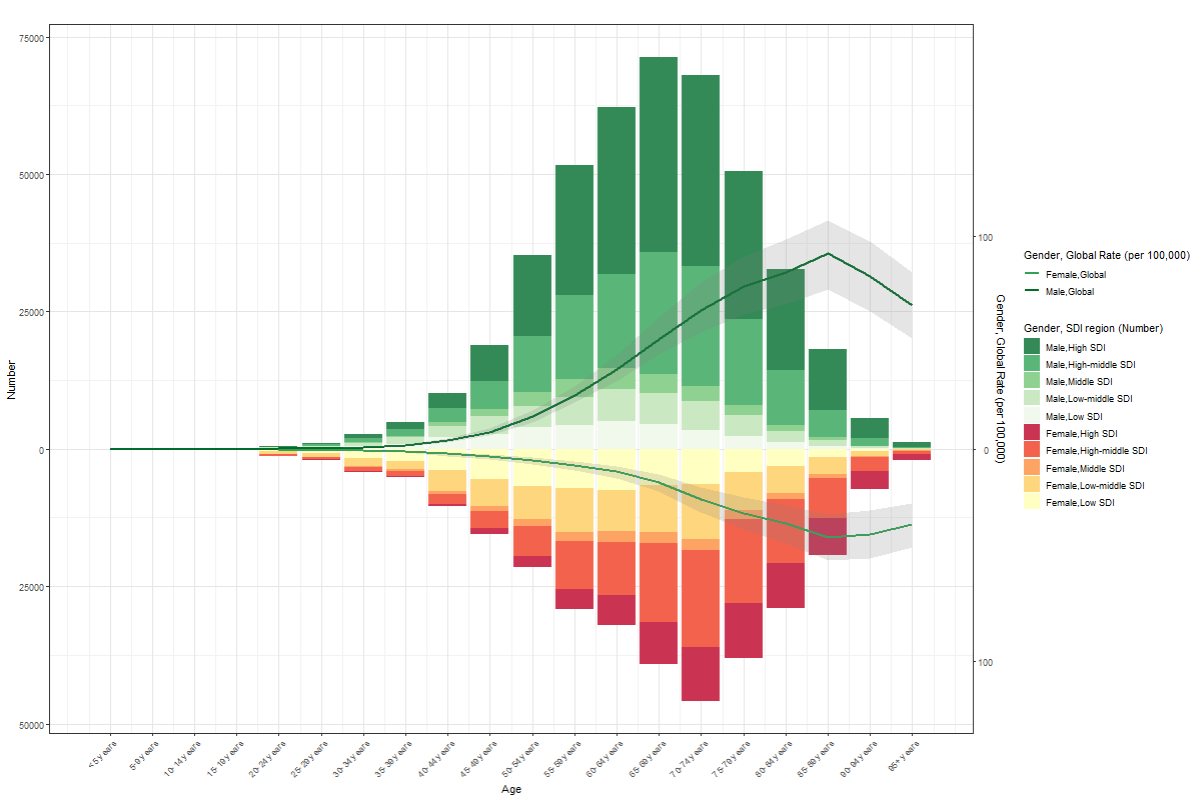
The age-specific numbers and ASIRs of OC by SDI regions in 2023. ASIRs – age-standardised incidence rates, DALYs – disability-adjusted life years, OC – oesophageal cancer, SDI – sociodemographic index.

A weak negative correlation was found between age-standardised DALYs rate and SDI (Pearson r = −0.18, *P* < 0.001) (Figure S1 in the **Online Supplementary Document**). High-middle SDI regions had a higher proportion of OC cases across almost all age groups for both sexes (**Figure 3**).

### National-level analysis

The ASPR of OC varied considerably across countries, ranging from approximately 0.9 to 48.3 per 100 000. Malawi (48.3 per 100 000), Madagascar (27.8 per 100 000), and South Sudan (27.6 per 100 000) exhibited the highest ASPRs (**Figure 2**, Table S3 in the **Online Supplementary Document**). Notably, all three are located in sub-Saharan Africa, aligning with our regional analysis. The most significant increase in ASPR was observed in Lebanon, with over 300% increase (Figure S2 and Table S3 in the **Online Supplementary Document**). Malawi had the highest ASIR (32.2 per 100 000), while Armenia had the lowest (0.6 per 100 000) (**Figure 2**, Table S3 in the **Online Supplementary Document**). These variations underscore a complex epidemiological landscape, potentially confounded by differences in screening, registration completeness, and diagnostic accuracy across nations.

In terms of ASDR in 2023, South Africa (10.0 per 100 000), the Democratic Republic of the Congo (9.5 per 100 000), and Lesotho (9.3 per 100 000) had the highest rates, all located in sub-Saharan Africa (**Figure 2**, Table S3 in the **Online Supplementary Document**). The highest DALYs rates were observed in Sudan (99.1 per 100 000), Germany (99.0 per 100 000), and Kazakhstan (98.9 per 100 000) (**Figure 2**, Table S3 in the **Online Supplementary Document**). The age-standardised DALYs rate increased most markedly in Ethiopia, India, and Gambia, all experiencing over 300% rise (Figure S2 and Table S3 in the **Online Supplementary Document**).

From 1990 to 2023, the most substantial decreases in prevalent cases, incident cases, deaths, and DALYs were observed in Kazakhstan, Armenia, and Georgia (over 50% decline) (Figure S2 and Table S3 in the **Online Supplementary Document**).

### Age and sex patterns

In 2023, the ASPR of OC increased markedly with age, peaking in the 75–79 age group. Males had higher ASPRs than females across all age groups. This male-to-female disparity was consistent across most SDI regions, except for low SDI areas (Figure S3 in the **Online Supplementary Document**). The ASIR demonstrated a U-shaped curve, peaking in individuals aged 85–89 years, with males consistently having higher rates than females (Figure S4 in the **Online Supplementary Document**). Compared to 1990, the ASDR in 2023 declined for both sexes under 84 years but was slightly higher in those older, with males experiencing higher ASDRs at most ages (Figure S5 in the **Online Supplementary Document**). DALYs also showed a downward trend in 2023 relative to 1990 for individuals under 74 years, with rates higher in males than in females (Figure S6 in the **Online Supplementary Document**).

### Risk factor contributions

Tobacco use was the leading risk factor for OC globally in 2023, accounting for 38.1% of attributable DALYs, followed by alcohol consumption (19.6%) and dietary risks (14.3%) (Figure S7 in the **Online Supplementary Document**). The contribution of these factors varied by region. East Asia had the highest proportion of tobacco-attributable DALYs (49.7%), while Western sub-Saharan Africa had the lowest (8.8%). Central Europe led in alcohol-attributable burden (34.6%). Central sub-Saharan Africa had the highest dietary-related burden (26.7%), while East Asia had the lowest (3.6%). The contribution of risk factors to deaths also varied across SDI quintiles. Tobacco use was the leading contributor in most SDI regions except for low SDI regions, where dietary risks played a predominant role.

### Projections to 2040

Based on current trends, the global burden of OC is projected to evolve significantly by 2040. The ASPR is projected to rise from 11.7 per 100 000 in 2023 to 18.8 per 100 000 in 2040 for both sexes combined. A sharper increase is projected for females (from 6.5 to 15.9 per 100 000) compared to males (from 17.3 to 22.6 per 100 000) (Figure S8 in the **Online Supplementary Document**). The global ASIR is projected to reach 10.2 per 100 000 by 2040. The age-standardised DALYs rate is forecasted to reach 234.4 per 100 000, reflecting a continuously growing disease burden, with significant regional disparities expected to persist. Across all measures, males are projected to have consistently higher rates than females.

## DISCUSSION

Our comprehensive analysis of the global burden of OC reveals a complex epidemiological landscape: an increase in absolute case number alongside declining ASRs over the past three decades. These findings are consistent with previous GBD 2021 studies [9–11]. The rise in absolute numbers can be attributed to population growth, aging, and continued exposure to risk factors such as tobacco and alcohol [15,16]. This highlights the importance of public education campaigns on these risks. Smoking is still the most important OC risk factor in 2023, as reported in previous studies [10,11]. For high- and middle-income countries, effective measures include strengthening tobacco control policies, such as increasing taxes, implementing comprehensive smoke-free laws, and expanding cessation services [17–19]. it should be noted that the GBD risk factor attribution is limited to a predefined set of exposures including tobacco, alcohol and diet. Several factors widely reported to influence OC risk including consumption of hot beverages, pickled vegetables, nitrosamine exposure, and certain infections are not included in the GBD comparative risk assessment framework.

Compared to the rise in absolute case number, the decline in ASRs likely benefits from health care improvements, particularly in these regions. The increasing availability of gastroscopy has enabled the detection and treatment of more precancerous lesions, reducing the overall burden [20,21]. More effective treatment strategies, including immunotherapy, have also contributed to better outcomes. In many high- and middle-income countries, a growing elderly population and improved diagnostic tools are detecting more cases, offsetting some of the progress from enhanced screening and therapies [22]. These findings underscore the importance of integrated prevention and treatment strategies.

Regional trends from 1990 to 2023 demonstrate a diverse global landscape. East Asia and sub-Saharan Africa bear the highest ASRs, while Latin America has the lowest. Beyond tobacco and alcohol, other risk factors contribute to the elevated risk of OC in East Asia, including hot beverage consumption, salted or preserved meats, leftover vegetables and nutrient-deficient diets [23,24]. In sub-Saharan Africa, particularly Eastern sub-Saharan Africa, which often termed the ‘African oesophageal cancer corridor’, the burden is disproportionately high [25]. Constraints within local health care systems contribute to delayed diagnoses and suboptimal treatment outcomes, aligning with studies emphasising that health care access is a crucial determinant of cancer outcomes in low-income settings [26,27]. Genetic predisposition can also amplify these risks, as evidenced by the familial clustering observed in high-risk areas.

On the national scale, China bears a disproportionately high share of the global OC incidence and mortality, accounting for over half of all new cases and deaths worldwide [28]. This is primarily driven by the high prevalence of risk factors like smoking and alcohol consumption. Despite advancements in national screening programmes, significant disparities in health care access between rural and urban areas persist, leading to high mortality and DALYs rates, particularly in rural regions where early detection is challenging [29]. This underscores the urgent need for targeted public health interventions in high-risk populations. Malawi had the highest ASIR, while India and Ethiopia experienced the most considerable increases, likely influenced by environmental exposures, tobacco and alcohol use, and limited access to early detection and treatment. In contrast, countries like Armenia and the United Arab Emirates have a much lower burden [30]. These regional and national disparities emphasise the necessity for tailored control strategies that consider specific socioeconomic, health care, and lifestyle factors, which requires strengthened collaboration among epidemiologists, policymakers, and clinicians. However, it is important to interpret national-level estimates with caution, particularly for low-income countries where primary data are sparse. In Malawi, Madagascar, and South Sudan, GBD estimates rely heavily on modelled extrapolations from neighbouring countries and limited verbal autopsy data, rather than comprehensive national cancer registries. Thus, the reported high prevalence and incidence rates may reflect both true epidemiological patterns and substantial modelling uncertainty.

Regarding age, the OC burden increases markedly with age, peaking in the 75–79-year group, with ASIR peaking in individuals aged 85–89 years. The different peak ages for prevalence *vs*. incidence reflect the fact that prevalence is a function of both incidence and survival. The later peak for incidence suggests that new diagnoses continue to accumulate into very old age, whereas prevalence declines after age 79 due to competing mortality and a smaller surviving population at risk. This pattern does not indicate a true decline in disease risk but rather the dynamic interplay between incidence, survival, and competing mortality. In recent decades, the burden of OC among older adults has grown substantially. However, older patients are frequently underrepresented in major clinical trials [31]. Data suggest that even in potentially curable stages, only an estimated 50–69% of older OC patients receive effective treatment, despite evidence that therapeutic intervention provides superior survival outcomes compared to supportive care alone [32]. There is an urgent need to broaden clinical trial inclusion criteria to encompass older adults and to conduct more thorough cost-benefit evaluations of treatment strategies tailored to this older population.

In terms of sex, males exhibiting significantly higher rates of incidence and mortality. For ESCC, which accounts for the vast majority of cases worldwide, the sex disparity is more parsimoniously explained by higher rates of tobacco and alcohol use among males. Biological factors may also play a role. for instance, androgens might increase the risk of EAC, while oestrogen could be protective [33,34]. Additionally, obesity and reflux disease, which are more common in males, are established risk factors [35]. Behavioural differences, such as women's potentially greater engagement in preventive health care, may also facilitate earlier detection and treatment. These findings indicate that age- and sex-specific health care strategies are needed.

In the correlation analysis between ASRs and SDI, high-middle SDI countries presented the highest incidence, prevalence and death, while middle SDI countries reported the lowest. Temporally, high-middle and middle SDI countries experienced declines in all ASRs, whereas low-middle and low SDI countries witnessed consistent rises. Several factors may contribute to the high ASPR in high-middle SDI regions, including more complete cancer registry coverage, an older population structure, higher diagnostic intensity and potentially elevated risk from urbanisation and lifestyle shifts. However, these regions also benefit from better health care access, more effective prevention programmes, and earlier detection, explaining the steeper declines in ASIR and ASDR. Conversely, the persistent burden in low-SDI regions highlights significant disparities in health care infrastructure, different age structure, screening availability, and public health interventions [36]. A weak negative correlation was observed between age-standardised DALYs rate and SDI. Given the very large number of data points, the statistical significance may not be clinically meaningful. Moreover, pandemic-induced disruptions may have exacerbated existing disparities in OC care, potentially explaining the continued high mortality and DALYs rates in some high-middle SDI settings [37]. This complex relationship suggests that socioeconomic advancement, while important, is not the sole determinant of OC control. The observed pattern should therefore not be interpreted as a simple causal effect of socioeconomic development without further analytical adjustment.

Projections to 2040 indicate an increasing disease burden. The rise in absolute numbers is driven by population growth and aging, with a more pronounced increase expected in low- and middle-income countries, highlighting an urgent need to enhance prevention and screening programmes. The predicted increase in incidence and DALYs reflects not only ongoing exposure to risk factors but also the substantial impact of OC on quality of life. Compared with those among males, the increase of OC ASRs among females are projected to be generally steeper. The possible reason for this phenomenon is the different relative magnitudes of risk factor exposure and gender-related OC management disparity [38]. Notably, our projection of a rising burden contradicts some previous studies using GBD 2021 data, which predicted a downward trend [10,11]. It is plausible, though not directly tested in this study, that the COVID-19 pandemic may have affected OC trends through disrupted care pathways, resulting in delayed or interrupted routine anticancer treatments, and COVID-19-related comorbidities and complications in OC patients may have heightened mortality risks, potentially contributing to an overall increase in OC-related deaths [39,40]. Furthermore, pandemic-related hospital admissions might have incidentally detected previously undiagnosed OC cases. However, the GBD 2023 data do not allow causal attribution of these effects, and the observed 2020–2023 patterns should be interpreted as descriptive only. Future studies using individual-level or interrupted time-series designs are needed to evaluate pandemic impacts rigorously.

This study has several limitations. First, the accuracy of estimates may be affected by variations in data quality and availability across countries, particularly in low- and middle-income settings where underreporting may lead to an underestimation of the true burden. Second, the GBD methodology relies on modelling and assumptions, potentially contributing to variations in data quality. Although the OC data for countries without cancer registries were modelled, this approach failed to address the issue of geographical approximation, as the distribution of OC exhibits clear geographical clustering. Thus, results should be interpreted as the best available estimates given current evidence. Third, our projections of OC burden to 2040 using Bayesian age-period-cohort models carry substantial uncertainty, particularly for the outermost forecast years. The BAPC model assumes that historical age, period, and cohort effects will continue in a similar manner into the future, which may be violated by unforeseen medical advances, changes in risk factor exposures, or emerging environmental hazards. Furthermore, the GBD study does not consistently report histological subtypes across all regions and years; therefore, our analysis focuses on overall OC burden but lacking subtype-specific estimates. Future studies should aim to address these limitations by improving data quality, refining statistical methodologies, and updating prediction models to better capture the evolving epidemiological landscape of OC. Despite the limitations mentioned above, as far as we can know, this is the first study to explore the burden of OC using the latest GBD 2023 database, providing valuable evidence for a contemporary understanding of this disease.

## CONCLUSIONS

Despite decreases in age-standardised rates, the absolute burden of OC remains high, with significant variations across regions, countries, and SDI quintiles. The findings underscore the need for targeted prevention and treatment strategies tailored to the specific needs of different populations, especially in high-burden areas. Strengthening health care systems, promoting healthy lifestyles, implementing tobacco control, and reducing socioeconomic disparities are crucial steps to mitigate the global burden of OC.

## Additional material


Online Supplementary Document
