# Supplementary material for: The global burden of oesophageal cancer from 1990 to 2023 and projections to 2040: a comprehensive analysis of the Global Burden of Disease Study 2023
Source: J Glob Health. 2026 Jul 10;16:04213. doi: 10.7189/jogh.16.04213 (PMC13351599; doi:10.7189/jogh.16.04213)
Supplement: Online Supplementary Document [file jogh-16-04213-s001.pdf]

Supplement to: Xing H, Xu Q, Hu M, Lei H, Wang Z. The global burden of oesophageal cancer from 1990 to 2023 and projections to 2040: a comprehensive analysis of the Global Burden of Disease Study 2023. J Glob Health. 2026;16:04213.

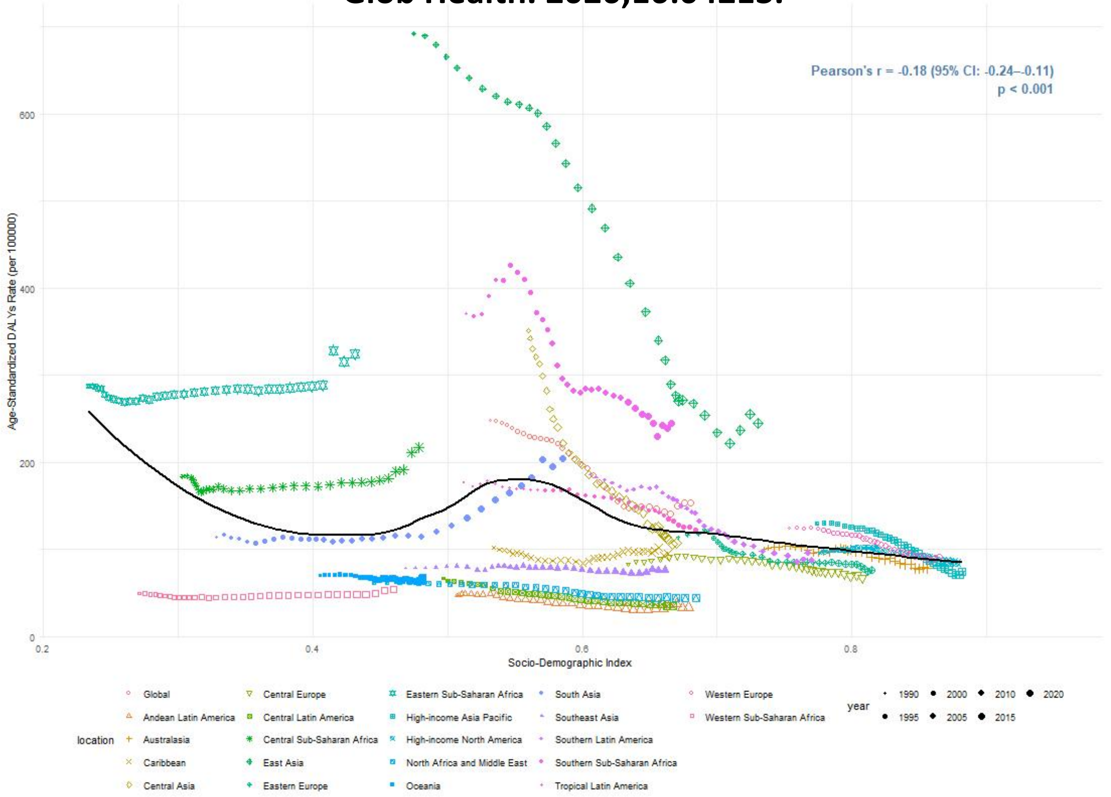

Figure S1 The age-standardized rates of EC DALYs globally and for 21 GBD regions by socio-demographic index from 1990 to 2023.

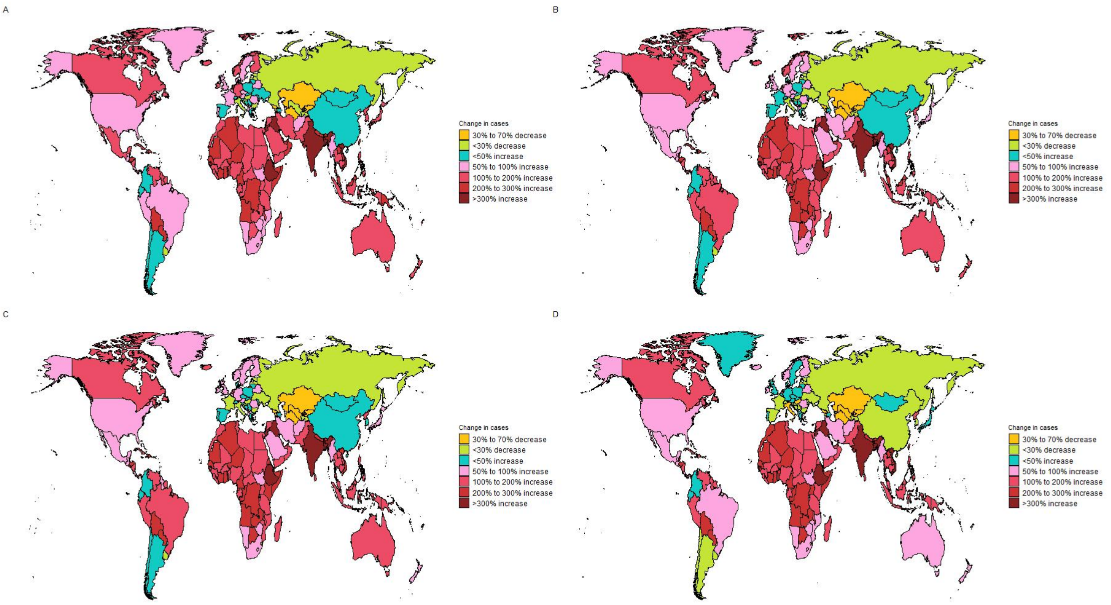

Figure S2 Change cases of EC for both sexes in 204 countries and territories. (A) Change in prevalence cases. (B) Change in incidence cases. (C) Change in deaths cases. (D) Change in DALYs. Figure 5 Age-standardized prevalence rates of EC by sex, age group, and socio-demographic index, 1990 and 2023.

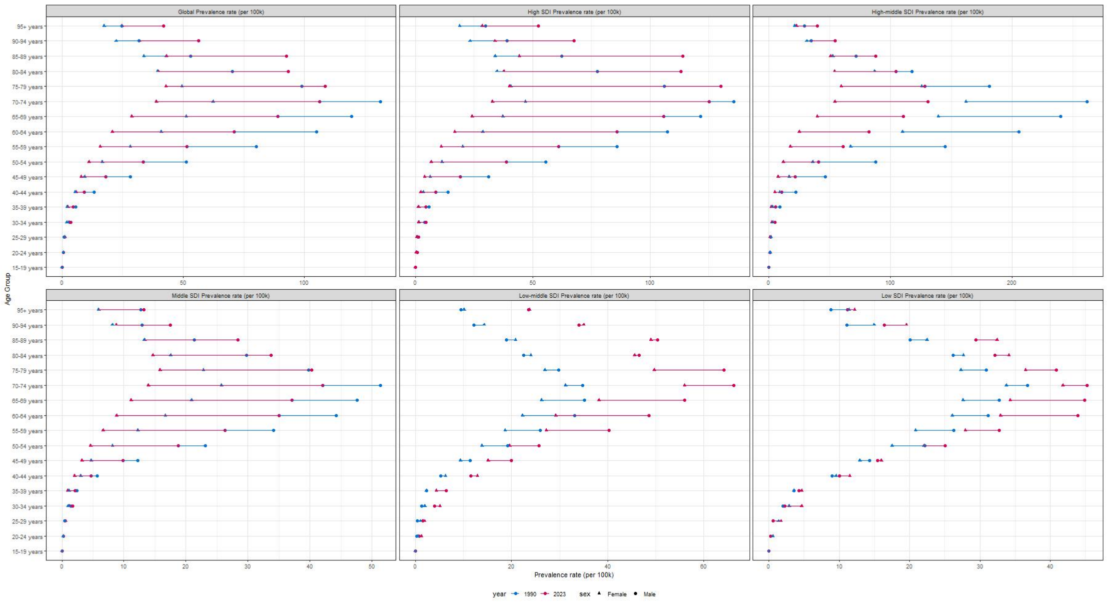

Figure S3 Age-standardized prevalence rates of esophageal cancer by sex, age group, and socio-demographic index, 1990 and 2023.

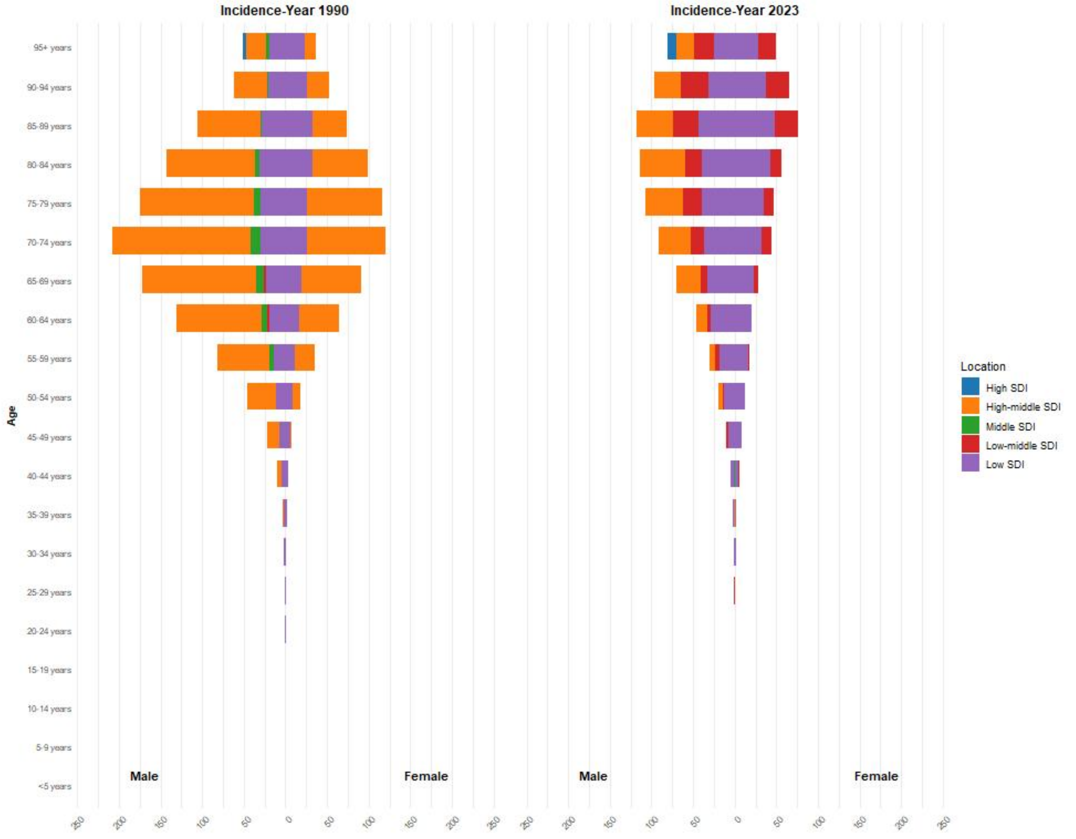

Figure S4 Age-standardized incidence rates of EC by sex, age group, and socio-demographic index, 1990 and 2023.

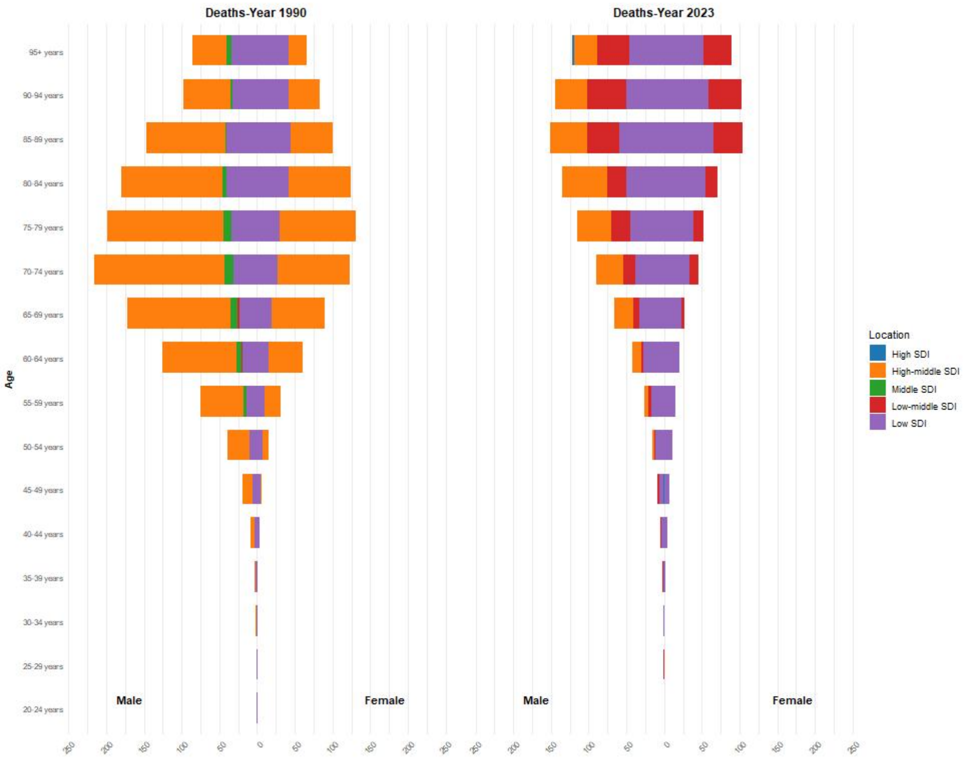

Figure S5 Age-standardized deaths rates of EC by sex, age group, and socio-demographic index, 1990 and 2023.

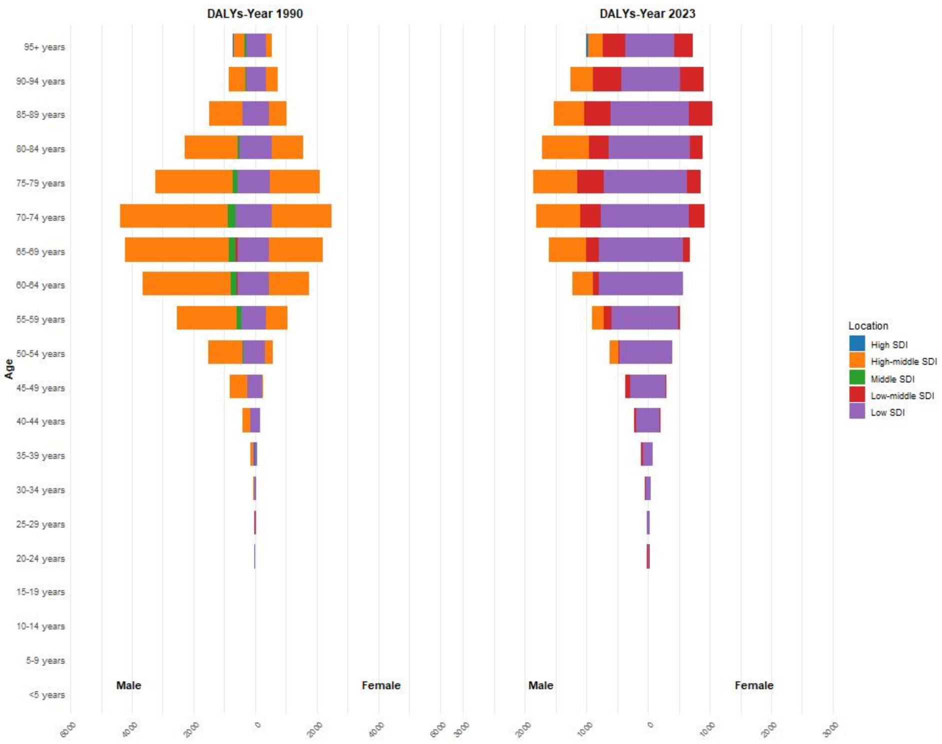

Figure S6 Age-standardized DALYs rates of EC by sex, age group, and socio-demographic index, 1990 and 2023.

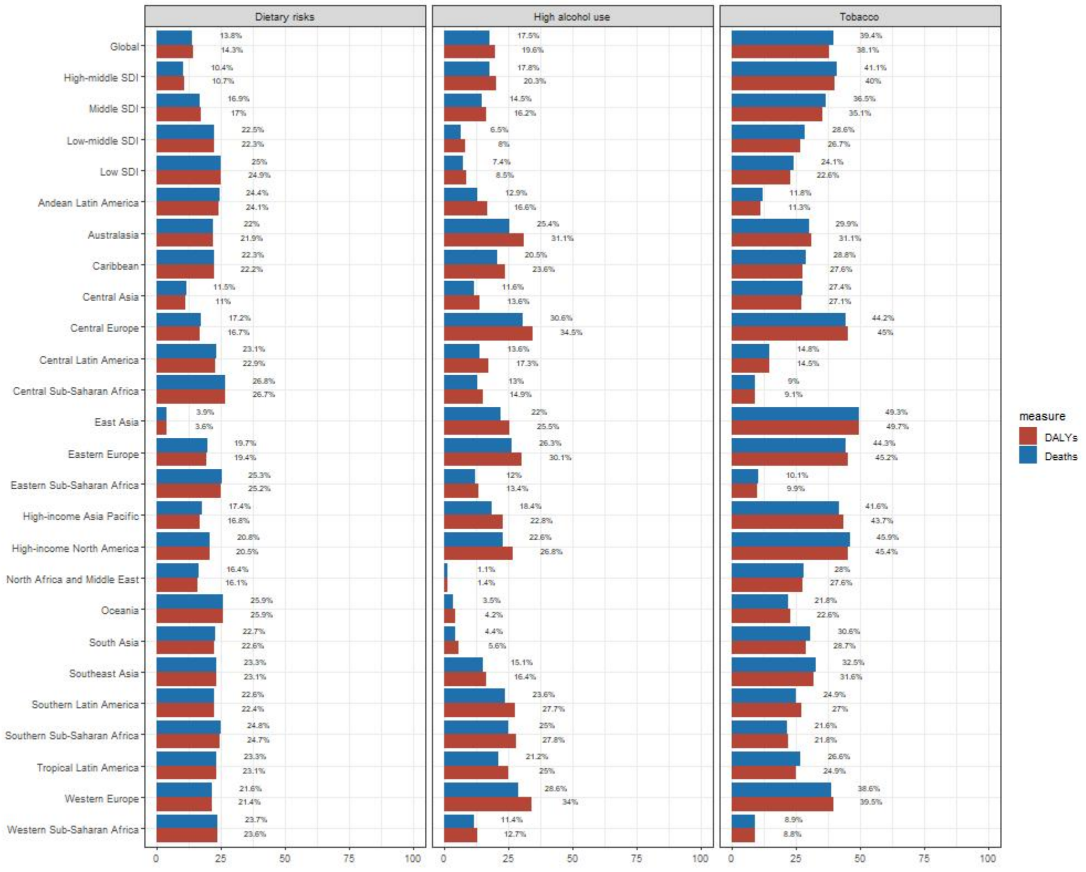

Figure S7 Percentage of age-standardized DALYs rates and ASDR of EC attributable to dietary risks, high alcohol use and tobacco.

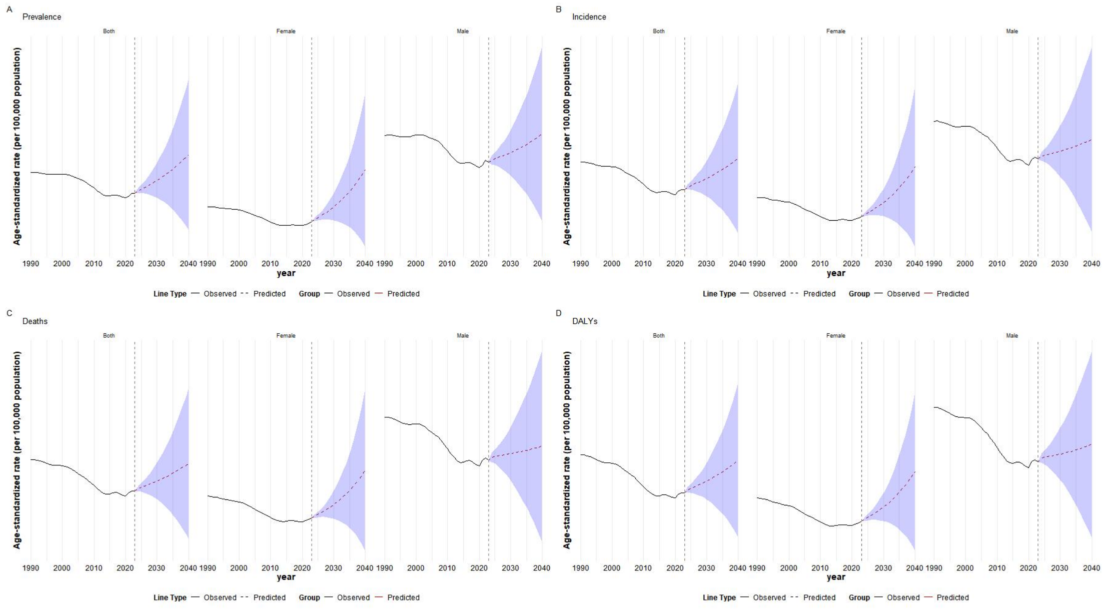

Figure S8 Future forecasts of global burden of EC.

Table S1 JoGH guideline checklist

1. Please list all papers published by each co-author in previous 3 years that were based on secondary analysis of a big data repository

Chen C, Xu Q, Wang T, Yuan Y, Hu Z, Li X, Zheng X, Lei H.Comparative analysis of liver cancer burden trends in China and worldwide between 1990 and 2021.Front Public Health. 2025 May 23;13:1513210. doi: 10.3389/fpubh.2025.1513210. eCollection 2025.
Xu Q, Li X, Wang L, Hu Z, Zhou W, Zhang Y, Lei H, Wu Y.Epidemiological Trends and Disease Burden of Tracheal, Bronchial, and Lung Cancer in China and Globally From 1990 to 2021: Results From the Global Burden of Disease Study 2021.J Evid Based Med. 2025 Dec;18(4):e70087. doi: 10.1111/jebm.70087. Epub 2025 Nov 12.

2. Please explain the key elements of your study design and the use of the available datasets that make your study an original scientific contribution

While previous GBD-based studies have described the EC burden, our study offers a novel contribution by integrating (1) the latest data spanning the full COVID-19 period, (2) forward projections to 2040 using BAPC modeling, (3) a comprehensive multi-level analysis across geographic, demographic, and socioeconomic strata, and (4) updated risk factor attribution. These elements collectively provide a contemporary, evidence-driven foundation for global EC prevention and control strategies.

3. Please list all publications that addressed similar research questions in the same dataset and indicate where you cited them in your paper

6. Morgan E, Soerjomataram I, Rumgay H, et al. The Global Landscape of Esophageal Squamous Cell Carcinoma and Esophageal Adenocarcinoma Incidence and Mortality in 2020 and Projections to 2040: New Estimates From GLOBOCAN 2020. Gastroenterology 2022;163(3):649-+. doi: 10.1053/j.gastro.2022.05.054.
9. Nimmagadda R, Majety S, Tummala N, et al. Geographic and Risk Factor Disparities in Stomach and Esophageal Cancer Burden Across the US: Findings From GBD 2021. Am J Gastroenterol 2025;120(10):S194-S94. doi: 10.14309/01.ajg.0001131040.05530.7f.
10. Wang ZL, Wei HW, Qi WF, et al. Epidemiological trends and projections of esophageal cancer in BRICS-plus: Based on the GBD 2021 database. Front Oncol 2025;15 doi: ARTN 161670210.3389/fonc.2025.1616702.
11. Zhang CC, Chen LZ, Xiu YQ, et al. Burden of esophageal cancer in global, regional and national regions from 1990 to 2021 and its projection until 2050: results from the GBD study 2021. Front Oncol 2025;14 doi: ARTN 151856710.3389/fonc.2024.1518567.

4. Please explain how you addressed multiple testing through an appropriately rigorous statistical threshold and indicate this in the methods section

Our study was primarily descriptive and hypothesis-generating, not confirmatory. Therefore, we did not apply formal multiple testing corrections across the many comparisons. Instead,

we focused on the magnitude and consistency of estimates rather than relying on p values. For the single correlation analysis between ASDR and SDI, no adjustment was needed as only one test was performed.

5. Please declare to what extent have AI chatbots been used in developing your paper and to which parts of the paper did they contribute

We declare that no AI chatbots or large language models were used at any stage of developing this paper, including study design, data extraction, statistical analysis, interpretation of results, drafting of the manuscript, or revision of the content. All aspects of this work were performed entirely by the human authors.

Table S2 Gather checklist

Item #	Checklist item	Reported on page #
**Objectives and funding**		
1	Define the indicator(s), populations (including age, sex, and geographic entities), and time period(s) for which estimates were made.	P3
2	List the funding sources for the work.	P15
**Data Inputs**		
For all data inputs from multiple sources that are synthesized as part of the study:		
3	Describe how the data were identified and how the data were accessed.	P3
4	Specify the inclusion and exclusion criteria. Identify all ad_hoc exclusions.	
5	Provide information on all included data sources and their main characteristics. For each data source used, report reference information or contact name/institution, population represented, data collection method, year(s) of data collection, sex and age range, diagnostic criteria or measurement method, and sample size, as relevant.	P3-4
6	Identify and describe any categories of input data that have potentially important biases (e.g., based on characteristics listed in item 5).	P4
For data inputs that contribute to the analysis but were not synthesized as part of the study:		
7	Describe and give sources for any other data inputs.	NA
For all data inputs:		
8	Provide all data inputs in a file format from which data can be efficiently extracted (e.g., a spreadsheet rather than a PDF), including all relevant meta_data listed in item 5. For any data inputs that cannot be shared because of ethical or legal reasons, such as third_party ownership, provide a contact name or the name of the institution that retains the right to the data.	P16
**Data analysis**		
9	Provide a conceptual overview of the data analysis method. A diagram may be helpful.	P4
10	Provide a detailed description of all steps of the analysis, including mathematical formulae. This description should cover, as relevant, data cleaning, data pre_processing, data adjustments and weighting of data sources, and mathematical or statistical model(s).	P4
11	Describe how candidate models were evaluated and how the final model(s) were selected.	NA
12	Provide the results of an evaluation of model performance, if done, as well as the results of any relevant sensitivity analysis.	NA
13	Describe methods for calculating uncertainty of the estimates. State which sources of uncertainty were, and were not, accounted for in the uncertainty analysis.	P4

**14**	State how analytic or statistical source code used to generate estimates can be accessed.	P16
**Results and Discussion**		
**15**	Provide published estimates in a file format from which data can be efficiently extracted.	P5-9
**16**	Report a quantitative measure of the uncertainty of the estimates (e.g. uncertainty intervals).	P4-11
**17**	Interpret results in light of existing evidence. If updating a previous set of estimates, describe the reasons for changes in estimates.	P12-14
**18**	Discuss limitations of the estimates. Include a discussion of any modelling assumptions or data limitations that affect interpretation of the estimates.	P14-15

Table S3 Disease burden of esophageal cancer in 2023 and trends compared with 1990.

Region	Prevalence in 2023			Change compared to 1990	Incidence			Change compared to 1990	Deaths			Change compared to 1990	Disability-adjusted life years			Change compared to 1990
	Value	Upper	Lower	Value	Value	Upper	Lower	Value	Value	Upper	Lower	Value	Value	Upper	Lower	Value
Afghanistan	4.042415841	1.957897135	6.58995688	0.750631652	3.240301284	1.461251598	5.219085224	0.578329057	3.50837265826507	1.57528554126332	5.63688140470959	0.533211943	75.5636103128109	33.8273695525976	122.093337989892	0.681106423
Albania	2.37905634	1.699420798	3.246088349	0.561177855	1.523605087	1.097157483	2.058590149	0.555149599	1.53909352356646	1.11137657049648	2.06610611546807	0.571218844	37.7175222147767	26.3240399493119	52.418772501335	0.409435363
Algeria	1.405879486	0.97392061	2.047484603	2.83553459	0.88796542	0.606114397	1.360254717	2.741107515	0.865541866149195	0.590669241863607	1.31328449660482	2.629902592	20.8280018401455	14.0800644187705	31.5184070047134	2.439550235
American Samoa	2.629251647	1.793368153	3.693314054	1.579811374	1.807960056	1.240276618	2.514028651	1.728553454	1.85567882991884	1.26948564556635	2.56749229976865	1.719449783	45.0251186404773	30.7767812029503	63.4267634692621	1.653487048
Andorra	8.697833058	6.030842914	11.96499649	2.064378177	3.89775974	2.705031556	5.278275762	1.795402672	3.15666257458514	2.23335554735129	4.26738748253707	1.572253935	83.0131782101522	58.6422311896799	113.320392723656	1.426465211
Angola	9.253900565	6.190684558	13.01200444	2.150184467	6.568349063	4.402937944	9.475678708	2.221560111	6.72727619532102	4.43964928095423	9.60033941318788	2.213379931	170.50219088967	113.146093430731	246.939420963705	2.16363219
Antigu	3.804952117	3.171842634	4.41159551	0.614606647	2.287161627	1.970836377	2.641575006	0.41691776	2.2563087911006	1.95152175822728	2.61057020949218	0.344401614	56.7268867148544	48.7791032728262	65.9520809303737	0.49639418

Argentina	6.64731695	5.794835132	7.498236474	0.082927846	4.269107282	3.80773818	4.744085301	0.059661644	4.30406598562474	3.81564203585628	4.81393934449035	0.064717912	97.1674604939739	86.9377198216854	108.090758419852	-0.040076215
Armenia	0.875943812	0.774808545	0.996754352	-0.602847322	0.570966819	0.501918313	0.654110164	-0.589304062	0.587724311823662	0.516950107186053	0.67117956893863	-0.572462986	13.5407523197617	11.8315620270632	15.4350368594224	-0.638137581
Australia	12.17815581	10.05142858	14.80609916	1.904576837	4.741348265	4.168548726	5.330872498	1.366534401	3.57500337410892	3.22821563824306	3.94047725536157	1.132655544	78.521843596768	71.3691224383054	85.6599469154552	0.860877198
Austria	5.883460888	5.036295159	6.889398006	0.987124519	2.907474075	2.528486758	3.311983796	0.720605077	2.55176163557545	2.23555400250282	2.88344686950824	0.605330951	59.9987359079131	52.9902663914351	67.2939437037786	0.411016498
Azerbaijan	7.66255135	6.246332007	9.315651007	0.237197518	5.266287939	4.247904519	6.360100202	0.260644597	5.42060575018164	4.31548560873753	6.58524387063716	0.268442387	130.208110064342	107.094248303521	156.374604203165	0.190189462
Bahamas	9.296786232	7.758440137	11.22661144	1.420052157	5.792300202	4.787170842	6.942247045	1.358612381	5.73261980407015	4.76460348954174	6.8373959017512	1.345469901	149.643695469284	122.992680549345	178.686208754267	1.270093588
Bahrain	2.873255089	2.054522716	3.811361759	3.009464958	2.053255482	1.470781295	2.656232942	2.52466246	2.08811872332226	1.50112769133217	2.72465656974057	2.262976743	42.9106488083614	30.8681611961157	55.8184650466171	2.299658622
Bangladesh	17.13547854	11.57455318	25.4438209	3.548148622	10.93289262	7.141324893	16.43107093	3.634382473	10.8449053961536	7.05494253812039	16.2783828445885	3.543016764	284.173123315025	184.475420313145	429.619722609774	3.332203341
Barbados	9.34421441	7.892080442	11.01141079	0.83245609	5.801786762	4.836879652	6.760064913	0.682222881	5.7409019655	4.8274648018	6.631787802	0.611485415	142.37050221	119.4557064	166.1583002	0.713167017

									8044	1474	86748		1408	5738	63818	
Barbuda	3.804952117	3.171842634	4.41159551	0.614606647	2.287161627	1.970836377	2.641575006	0.41691776	2.2563087911006	1.95152175822728	2.61057020949218	0.344401614	56.7268867148544	48.7791032728262	65.9520809303737	0.49639418
Belarus	5.930635618	5.261708233	6.714559416	0.907437084	3.285323033	2.900730332	3.69186287	0.743634142	2.97016285071802	2.64424878331033	3.32935903420977	0.641875027	84.8916102906208	75.6403472292807	94.897690005776	0.637523235
Belgium	9.240785744	7.825250589	10.57304288	0.88602512	4.681834516	4.051443146	5.234058181	0.693821706	4.18892831113793	3.61987339246162	4.65967892269543	0.61737983	96.8357616749013	84.1491661299873	107.200927089472	0.42183111
Belize	3.642852533	3.08187603	4.301248119	2.878352678	2.28715636	1.941703143	2.665804803	2.584639389	2.29540087337551	1.93402477645783	2.67756016450233	2.44500919	58.6411872865486	50.011183921855	68.9821813807546	2.760031965
Benin	5.718802622	4.160628564	7.76217518	2.250567605	3.86105355	2.655963543	5.27058623	2.014340319	3.85788473335308	2.65120050350379	5.26697163678918	1.914020715	103.728027025813	72.7722665394734	140.900851040764	2.162077623
Bermuda	8.612071551	7.157063082	10.14798978	0.547414215	4.526080854	3.850640013	5.213576521	0.385998124	4.13933971386577	3.54376189810047	4.75274473845605	0.326096634	100.098876353994	84.8613141876036	115.795048471794	0.164098634
Bhutan	9.281482101	5.913643902	13.76335637	2.435886496	6.202680572	3.743063737	9.434138198	2.508424061	6.1100809058192	3.69670260341823	9.21831792082298	2.486694318	166.914076552275	100.380063852394	252.963648643153	2.406797366
Bolivia	3.716257328	2.619350702	5.200743075	2.622416792	2.706006964	1.810163867	3.875073134	2.944661772	2.83316655856179	1.89613332036967	4.07125726493224	2.922687595	64.4216688793803	43.8113435080787	92.5236762011527	2.672689877
Bosnia	2.9798	2.3754	3.4790	0.009585	1.9466	1.5861	2.2735	0.084506	1.9469	1.5896	2.2838	0.120686	49.113	39.584	57.536	-0.04843

and Herzegovina	85361	13962	42448	578	42239	44251	43632	664	1400251803	2472611906	0810799793	477	9568863202	1228105168	4018646533	1514
Botswana	9.884854082	7.089387621	13.52027789	1.944480198	7.991810742	5.805328193	11.2229763	2.330794818	7.87077789608097	5.74272082964015	11.158730017132	2.291627401	214.875861516596	153.762844256484	298.216757826143	2.292693832
Brazil	7.228844783	6.714878441	7.711833603	0.942030581	4.802482499	4.468335318	5.108935171	1.064631262	4.74424508900526	4.39952550554148	5.04311854932241	1.077792194	122.043070992749	113.962678980302	129.216979154587	0.911628831
Brunei	3.754716982	2.856986633	4.949140441	2.469570386	2.053156721	1.601109065	2.572104213	2.007282308	1.8816327880181	1.44434452665608	2.36071914765183	1.77047723	45.6455292225954	34.8932871210842	56.6286261405128	1.876597942
Bulgaria	2.817197429	2.443810919	3.219112827	-0.162362024	1.746036453	1.522347483	1.970580083	-0.164059414	1.71393037642475	1.49428969223586	1.92500145245775	-0.155657369	47.3987137851069	41.5254408815751	53.5316247876777	-0.201659223
Burkina Faso	3.735588443	2.732087897	5.156990098	1.504365461	2.744800451	1.997314939	3.722891935	1.515869692	2.83938544173109	2.05767449237131	3.83291500209857	1.504408051	68.9891674616836	50.5248512399438	94.1236244594367	1.52876681
Burundi	18.62529689	12.37809528	28.20264484	1.78909783	12.31640145	8.1227695	18.57247408	1.956417876	12.2149008715151	8.05852901629842	18.4551588374044	1.891493434	345.446454689877	225.047611717951	521.869177219387	1.981652914
Cambodia	3.187016767	2.292836295	4.567999253	1.314999173	2.043848214	1.463613369	2.966631499	1.570801588	2.03805443387916	1.46104200547757	2.95936414767301	1.674091497	53.6947384237524	38.348706398007	78.3422225070059	1.216352725
Cameroon	4.655437714	3.123239424	6.628213936	2.179897576	3.348673465	2.266116199	4.598205245	2.298823861	3.3930693944	2.3011803957	4.641126275	2.281705896	87.353472857	59.00791955	120.4668456	2.252382387

									1774	2872	88425		6117	82791	89671	
Canada	9.827717747	8.57684667	11.30354525	1.622513045	4.487232814	4.0878104	4.945942208	1.358566434	3.77808065188965	3.50370072616432	4.05540197628922	1.245889939	85.6759049182523	80.3477856038757	91.7168062656376	1.01517283
Cape Verde	16.43767141	10.86192907	23.21363422	1.461837981	11.80091137	8.166836558	17.09566286	1.486566726	11.5926065094288	8.07466740870106	16.568608168905	1.270425401	315.153911646745	215.102494068039	466.325353313364	1.895758584
Central African Republic	8.87831839	5.430045835	13.56476853	1.064533459	6.214447871	4.059297095	9.115773754	1.28327097	6.46274879170765	4.21095663297341	9.53840960642439	1.286443527	161.225803181595	105.154293693259	237.743347005382	1.268759221
Chad	5.2732398	3.739723261	7.346586534	1.579166299	3.872264102	2.788698868	5.204721388	1.52301246	3.95837495347228	2.85148980881161	5.29301215196578	1.464806559	100.681064120228	72.2938275713575	135.695781987511	1.656815405
Chile	4.797663507	4.317075168	5.299353069	0.161228441	2.991997743	2.741414081	3.232188577	0.072300554	3.03397099886782	2.74982155543637	3.26524068128253	0.06104307	59.792121310914	55.6722274407278	63.6452032555597	-0.098980822
China	19.35827869	16.92705436	21.76915705	0.183459919	11.45095864	9.831288477	13.09949516	0.099506583	10.9462355360424	9.34678026637489	12.5452249418289	0.070351084	244.501000497829	216.477817368037	278.63477519025	-0.104010345
Colombia	2.482696848	2.235971328	2.777655786	0.281899987	1.58311691	1.427812051	1.757684985	0.238744677	1.61317857336906	1.44934177131005	1.80662188077749	0.242175458	34.7489218422514	31.6658069425293	38.4557810812091	0.07373059
Comoros	8.60558642	6.064041621	12.11425243	0.912472895	5.561764018	4.007637108	7.723864066	1.065554421	5.58062630255043	4.00678383122344	7.74270421327367	1.123528939	148.375718659015	107.119825055555	206.713631686604	0.935936267
Cook	6.2709	4.1839	8.9355	1.263889	3.8811	2.6859	5.5439	1.395411	3.7383	2.6009	5.3042	1.353178	91.789	63.096	132.81	1.124179

Islands	99968	75509	17328	764	20832	51585	94984	073	6793552595	8346607546	6164983235	531	5655446287	9041961126	8008024223	344
Costa Rica	2.619407173	2.21710404	3.092128426	0.959277161	1.593749808	1.357890593	1.850045253	0.78629235	1.59940564241297	1.38291498516647	1.84094357875532	0.727309382	34.96636355	29.8029621899009	40.5604186244753	0.715478456
Croatia	4.385616357	3.863701628	4.985435109	-0.128479275	2.645041499	2.337204526	3.018800145	-0.110531347	2.57135722145861	2.28494743874528	2.93730342747999	-0.089890739	66.1488647039212	58.6606624967789	75.3531938833092	-0.239461783
Cuba	7.622983226	6.673633577	8.804952916	1.241214608	4.680283397	4.013927315	5.384588775	1.176976003	4.49043862094021	3.86954880398242	5.13227867031565	1.083637901	114.066041535971	96.6687003799496	131.12622610103	1.163623321
Cyprus	3.77256516	2.76693162	4.980873453	1.870054255	2.045554855	1.468289599	2.698269573	1.29351321	1.90084713594205	1.37086479258619	2.51475258048269	1.068406555	40.3422069434586	29.3273432361217	53.208919473436	0.9295735
Czech Republic	6.285692306	5.65758061	6.979672658	0.750261887	3.233691651	2.983570292	3.507638724	0.525227764	2.89498232683496	2.69332938272919	3.12418684633427	0.437988623	74.4534249890242	68.9469573031627	79.9574722276411	0.295627654
Democratic Republic of the Congo	12.79778043	7.681861533	20.13727174	2.23180548	9.253760666	5.76457611	14.32120617	2.370887742	9.5326872306509	5.92823366525271	14.67823242 66188	2.400571348	238.278809776632	153.519096557231	375.420596672151	2.273048263
Denmark	10.77520833	9.310161215	12.38786742	0.639725955	5.615023344	4.935731732	6.378382364	0.430673456	5.07476615107037	4.48511470268175	5.69808062885418	0.346856341	112.478015171587	100.313002868153	125.723548239095	0.151850751
Djibouti	17.538	10.653	26.666	2.699678	11.131	7.0396	16.545	3.019326	10.989	6.9146	16.230	3.103570	309.51	196.59	458.02	2.789266

	94847	24332	09469	216	53725	76822	29856	188	4751863041	7330288087	2957323885	836	3173153841	3691996387	2243742928	374
Dominica	5.662937106	4.238354571	7.947769881	0.474672477	3.687018741	2.725879669	5.18627156	0.409410218	3.74686336484172	2.76400392931572	5.26706118681665	0.393342849	93.5475914979467	68.5683657672385	131.216405837395	0.430823185
Dominican Republic	4.146343439	3.033027793	5.700609641	1.879606675	2.877585451	2.1073825	3.950296691	2.039862784	2.93792547219144	2.14586535785677	4.04746023528145	2.035974101	72.5747400638588	52.6413096345583	99.9929285517918	1.961437035
Ecuador	1.550713286	1.380591453	1.739397953	0.325651864	1.041526571	0.93195367	1.172900633	0.29704517	1.09562559635682	0.972558910963104	1.225919942 9427	0.301496338	24.0093280550949	21.5506042839384	26.8888943197401	0.195256594
Egypt	1.709828183	1.331681821	2.225798828	1.699657477	1.215762938	0.930013257	1.571161876	1.827029778	1.24116307741852	0.934068154731103	1.61714204723706	1.803552331	28.1553613857364	21.1268834842301	36.2810659196468	1.477060409
El Salvador	3.324591704	2.606528915	4.056882988	0.987521643	2.238064632	1.732929935	2.790002035	1.036757292	2.30113338634889	1.77914625424144	2.84458414044496	1.04476394	52.6755260203438	40.6337671524849	67.1601239645367	0.847832796
Equatorial Guinea	12.61245667	7.758508821	18.88237367	2.274398611	10.61895357	6.573441338	15.08918641	2.858295573	10.6517322195605	6.63202069678469	15.0340639634653	2.741880739	279.758120517126	168.185531328594	401.519814188723	2.899923373
Eritrea	18.56598915	13.06260388	26.38382257	1.891980899	11.88428875	8.265019938	16.77034088	1.98162357	11.7431273772708	8.16378110229937	16.5140059324252	2.00697249	335.80805409828	233.547129904702	473.970772161185	1.886475026
Estonia	4.770103323	4.127151386	5.376946603	-0.023398141	2.645867163	2.35795041	2.937146002	-0.126660475	2.41786125492521	2.16424499897804	2.67439394630924	-0.169516005	62.0523149892505	55.1753050667647	69.1950718661276	-0.255395098

Ethiopia	12.0459934	8.395716078	16.85760863	3.971868723	8.102591139	5.946960727	11.00838566	4.550011995	7.9967377101385	5.8109316020417	10.76479945 97155	4.534092643	223.032418077576	164.8649596	306.40117297058	4.286552148
Fiji	4.203263812	2.864029956	5.918828451	1.569849765	3.084360737	2.146729062	4.199410589	1.895064234	3.19417438359151	2.2062450655872	4.40103468018082	1.934395362	76.4352643451426	53.2437124791448	105.453336849223	1.699335525
Finland	9.855108053	8.094053105	11.82106929	1.253830638	4.079083776	3.515853886	4.714200021	0.900607488	3.19765625809231	2.77634737587101	3.5941039618527	0.726755539	75.1246218479598	66.0614878140504	84.3795190028419	0.544227572
France	15.137767	12.0479213	18.20907895	0.597903079	5.396528034	4.671414513	6.075830123	0.10610938	3.78806231948626	3.32826429400217	4.18362818781956	-0.121867949	91.8943613855832	80.8834969635883	101.176991034474	-0.261517604
Gabon	9.592048878	6.2617732	13.6032828	1.551375917	6.747651079	4.414015551	9.313967188	1.685597797	6.86895505405942	4.46510110222814	9.52287611061606	1.684315014	173.444072905937	113.753726618398	240.322649783288	1.617267605
Gambia	1.647214479	1.184210035	2.315164687	3.262296005	1.126418291	0.804109898	1.528069946	3.466245877	1.13544120561951	0.815867050140909	1.54835533861311	3.492762696	29.5903442476888	20.8262878179014	40.185423042866	3.370814335
Georgia	1.583302608	1.366041838	1.865580485	-0.558124455	1.063658425	0.899889452	1.260565931	-0.536030395	1.07385802650445	0.909358473312514	1.2732810030948	-0.526332279	26.7217012095596	22.4587508864965	31.7591798823969	-0.567412221
Germany	10.97981258	9.536946471	12.89064904	1.313614994	4.992499798	4.559252839	5.51068446	0.98730512	4.06031587114656	3.74312270058459	4.37524682333895	0.805440649	98.9983989696982	90.5916303260753	107.26202866064	0.496746413
Ghana	4.552487366	3.301702169	6.039594828	2.237706278	3.071898309	2.25109477	4.215467956	2.306817566	3.0296349737	2.2114581965	4.154049244	2.293131332	84.191376544	61.08714394	116.7689360	2.26294233

									511	5042	40526		0269	45551	6985	
Greece	3.425991563	2.910576315	4.022195038	0.279113386	1.741830315	1.493951723	2.001617475	0.150932101	1.56902015818852	1.35444020264825	1.78859378609923	0.114716744	38.3030866818193	33.1857397574061	43.7115379093125	0.094072888
Greenland	21.86418795	16.51615905	27.69043299	0.56351285	15.21261015	11.49204438	19.56762617	0.566286808	15.8481086811216	12.1168158593986	20.3492044243986	0.588199385	319.29847871742	240.637293509283	411.603004944389	0.345649997
Grenada	7.447748671	6.138626048	8.702330803	0.674644355	4.782024885	3.961175233	5.716537832	0.570763526	4.6992378468563	3.87152528608988	5.61635446013752	0.509026329	126.915183788112	105.460163365689	151.694375446972	0.627692439
Grenadines	3.887829119	3.19902108	4.622555777	1.138770014	2.431743153	2.005492875	2.945106401	1.027508109	2.38392199935824	1.97289354251639	2.88017866265206	0.963506487	65.4220345224292	53.6703086962742	79.7597310091438	1.103293061
Guam	3.05110005	2.562983505	3.51856397	0.68003389	1.727243352	1.46756974	1.95287481	0.603762296	1.66435069360822	1.42843264897692	1.88346708855703	0.593525002	42.7592735628749	36.2679120424049	48.5857337459386	0.463138774
Guatemala	2.370169245	2.090008076	2.69574944	0.818608915	1.667197585	1.427494419	1.902055527	0.759146439	1.78194220756086	1.52062536623352	2.0374299951998	0.789831163	39.039451011733	33.4937473405588	44.2746447851431	0.608555775
Guinea	1.42045777	1.01737257	2.01463616	1.276602903	0.962154309	0.671283841	1.371590613	1.191279727	0.969563259579894	0.680079107017846	1.38570335943389	1.151457382	25.719241634752	17.9583303207605	36.77264181	1.272749852
Guinea-Bissau	4.306173564	3.099407898	6.003025433	2.156651496	2.996718993	2.146235805	4.10385099	2.047423086	3.04279406623419	2.17527280384132	4.16411532423577	1.958791195	79.3626269328997	56.0291405402683	109.127396904268	2.194332346
Guyana	3.2336	2.7005	3.7710	1.067537	2.1154	1.7609	2.4740	1.006285	2.1027	1.7606	2.4676	0.972241	56.899	47.275	66.563	1.016880

	39778	92846	40562	303	02734	95809	07293	888	48493	9825415404	8381368126	235	0144681114	1128866379	8849502763	557
Haiti	6.896299875	4.792185225	9.778622236	1.778893942	4.817073035	3.414665952	6.714512848	1.893866724	4.92664429645933	3.50237804011503	6.8646900532704	1.861683771	126.513562472323	89.3557931855215	179.371643548537	1.89417345
Honduras	1.177142202	0.829458918	1.717591841	2.027890817	0.972762541	0.660661628	1.390374236	2.486198784	1.08543389652881	0.735296432775637	1.55803428002036	2.63664833	21.8377445700384	14.9157793576216	30.9171948689008	2.161775674
Hungary	4.472901507	4.025073171	4.845916412	-0.223568994	2.779370667	2.518579488	2.975742013	-0.188541466	2.71084202675274	2.48119952446626	2.89986137819603	-0.16401724	71.2032714926188	64.6204137734237	76.4221276090581	-0.311150465
Iceland	11.32055495	9.360710504	13.8682445	1.813105375	5.198136378	4.359908144	6.168763343	1.321597769	4.41857383144917	3.80811860287908	5.14128164682669	1.115697803	101.755225189769	86.7080655891279	118.557420371931	0.971909114
India	11.18462263	8.726236001	14.10628897	4.187489363	7.772222637	6.091103569	9.763237823	4.48820349	7.95305669738176	6.18764304848595	9.95455408737159	4.603877703	195.916857139474	151.7020886	248.236029031329	4.032837531
Indonesia	3.791544637	2.578187602	5.560929018	1.745610161	2.31637263	1.581066262	3.318825032	1.683837398	2.22353187161832	1.50652575562201	3.17028886934015	1.603425384	63.8967028797808	43.3597253103348	90.7938118432504	1.66903417
Iran	6.62564562	5.172271767	8.399053246	1.300714636	3.91967895	3.134459682	5.018239472	0.96420194	3.89979414812775	3.09333047376153	4.93849548420697	0.890338157	81.6827610204504	64.9111815996717	103.215970845097	0.72424439
Iraq	2.889097716	2.095025573	3.865343071	4.154610084	1.696270775	1.217215276	2.221662255	3.588305187	1.63293669404099	1.178968797	2.17944831629114	3.19442112	40.6088718056922	29.4824868455891	53.2151186894035	3.353303206

Ireland	15.23144622	12.89645599	17.9387604	1.47587834	7.26536147	6.268594273	8.113818519	0.891714372	6.28142629445536	5.49905871999438	6.92523241080674	0.670360538	137.948564985674	121.929187132366	151.899239094195	0.570971384
Israel	3.110764074	2.590207329	3.703167216	1.554196231	1.611265709	1.376307281	1.864390501	1.108437816	1.47221657185489	1.26890905132833	1.69066982315442	0.950453182	33.0174346313375	28.3854967430501	37.8542312938589	0.855421931
Italy	3.643635525	3.077522567	4.318184276	-0.09782949	1.784667719	1.51657407	2.037888579	-0.187505408	1.58276764887731	1.35126864576766	1.79076925375874	-0.215476451	37.053996378614	32.185765294386	41.6702750993488	-0.335969218
Ivory Coast	1.196415395	0.870605722	1.705032543	2.269905726	0.796279748	0.561060269	1.070614664	2.320100818	0.791940993971973	0.558206465933971	1.0685501391374	2.297308221	21.7511545387578	15.2193184204553	29.2446938954233	2.308484279
Jamaica	5.371261473	4.488829636	6.146325329	1.007548797	3.475711979	2.937070297	4.109887462	0.892896978	3.4428791187228	2.93900081448796	4.08147439916912	0.81216616	87.8753548393933	74.0592856263128	105.058642605089	1.021742079
Japan	20.20436723	18.02382309	22.02836467	1.348529288	6.214773127	5.607800323	6.664943576	0.985002241	3.86276737330638	3.49558265082793	4.14634645388655	0.765310837	84.0575331683498	77.2522465154178	89.9755173048454	0.313092588
Jordan	2.562813941	1.863173342	3.456088116	4.214931702	1.588984623	1.175318748	2.141483171	3.92314421	1.54288530535537	1.12164199456788	2.10020404809323	3.678435023	35.0339923354626	25.505381439271	47.9027862106631	3.324770356
Kazakhstan	5.985321039	5.518465207	6.458604706	-0.653634478	4.006411744	3.654405726	4.314151737	-0.663121924	4.06799776241198	3.7092888845923	4.39594759292384	-0.66736671	98.9040848061178	91.181060603572	106.028209306278	-0.672240027
Kenya	21.67793014	15.61577381	30.45137477	1.82194427	15.29686178	10.89472714	21.25984017	1.983562388	15.233358225	10.938753126	21.27432251	2.022848349	408.98732820	290.6520245	564.9577899	1.872211702

									9969	4284	35213		6808	41571	49242	
Kiribati	4.772290771	3.080559436	7.140267868	1.391256026	3.081331131	1.969561769	4.453851917	1.490194533	3.06847628012874	1.96952561077225	4.4363069330475	1.450218343	85.7859814301741	54.0749690201316	124.255365870541	1.514357468
Kuwait	2.065219563	1.698667146	2.431583395	1.431895363	1.07776161	0.918541233	1.234869884	1.008237043	0.995910503946405	0.860396208482774	1.13702517838442	0.802243881	20.1776611716853	17.581244528125	23.0029179284579	0.783046978
Kyrgyzstan	3.829791765	3.435527861	4.218807172	-0.423258868	2.739414689	2.448898898	3.017452851	-0.422776096	2.86375985280016	2.57523590292749	3.15869422319628	-0.425140861	64.7501247780824	58.059120963243	71.1635623777114	-0.449556075
Laos	4.812089662	3.401412723	6.737203616	2.233120688	3.12103083	2.235625371	4.360977477	2.123418939	3.07793356521299	2.19741670517406	4.30383376703611	1.995916229	83.1806807129831	59.8428995442528	117.594097000989	2.148991575
Latvia	5.658396303	4.788339064	6.670128974	-0.050281437	3.298369183	2.842983142	3.82025974	-0.100757691	3.09194773799828	2.67992968727822	3.5798943672359	-0.122441652	83.8518871106992	72.2172434228805	97.8598609581446	-0.174086485
Lebanon	1.977721535	1.447845277	2.638141825	4.226177201	1.037917171	0.769219203	1.342847431	3.598380435	0.978871637597685	0.726237837760441	1.25208227026536	3.354976944	23.5857822511046	17.5494105468982	30.602448323684	2.962690269
Lesotho	11.80606508	7.81520753	16.6514216	-0.027431945	9.224584912	6.526711273	13.06895414	0.179369707	9.30470218062246	6.58603738245441	13.1234182549942	0.193103826	248.992488957013	177.316761755801	358.236041268235	0.161899235
Liberia	5.567489546	3.794411227	7.916267517	1.98902002	3.832074695	2.603964607	5.652899045	1.811212199	3.81766314216907	2.61994952577366	5.63211066177609	1.700353877	103.598572833304	70.0952640434776	151.708649993122	1.984014758
Libya	1.7150	1.0967	2.5191	1.888720	1.1125	0.7314	1.5918	1.798798	1.0826	0.7342	1.5397	1.715535	28.046	17.821	40.501	1.756121

	72098	17826	92198	927	34843	53782	73239	208	754014581	52564043072	2812439992	233	634879982	9881244093	1902294944	65
Lithuania	6.658121397	5.875472485	7.499785593	0.304854103	3.816262119	3.360165902	4.281440752	0.238743954	3.54510515223711	3.13524733618039	3.9745129816235	0.203466981	98.2376913009389	87.1124975208545	110.750926732732	0.147441205
Luxembourg	8.164133604	6.64887475	9.883816109	0.983849796	4.040429785	3.390487449	4.719981932	0.639008948	3.56597307524094	3.03605133857481	4.12719924957425	0.503662488	82.2237848648959	69.3250942197715	95.568009327342	0.358187962
Madagascar	27.77002864	19.64441384	38.62399065	1.160097402	19.77539949	14.23526473	27.47363573	1.207087964	20.1252583014564	14.4710088054606	27.9279730242137	1.215722257	525.418576277366	379.105062722348	732.346200609395	1.141822412
Malawi	48.25320429	33.96105176	66.95964093	1.758091897	32.22761105	23.22990868	43.9356483	1.852438385	31.3161040122603	22.5556851136886	42.4447108332498	1.86802971	928.978960241919	668.038101098401	1275.49303436077	1.768890496
Malaysia	3.005023089	2.484214423	3.680641195	2.155416221	1.901527552	1.585631991	2.300648026	2.013761737	1.87205624993477	1.57163091581093	2.26073192122774	1.909244672	45.731411616018	38.6748846546888	55.22726951	1.84930168
Maldives	3.889254912	3.058785638	4.983391339	2.621524245	2.565756572	2.022388524	3.24442181	2.406154053	2.58391194992819	2.0426139510707	3.26053591694412	2.341227268	51.593338542205	40.6456470474555	64.6503498534261	1.73081964
Mali	4.384668593	3.232862374	5.922201577	1.925789076	3.035524049	2.178470176	4.070009759	1.947897425	3.04295869807155	2.18459768918717	4.07541542811953	1.9301131	82.0300429670085	58.7405927722841	110.718534333863	1.944098978
Malta	4.723301698	3.890384587	5.736848283	1.162310042	2.395290184	1.998771788	2.81771189	0.857202266	2.15315490226601	1.81186525897521	2.52599205270742	0.748702383	49.9512998873343	41.8226387836682	58.9055869490068	0.560854169

Marshall Islands	4.890095783	3.32638556	7.044877425	0.704699009	3.925845073	2.662429716	5.577925474	0.793169673	4.10372091709245	2.79052212997345	5.78693128440699	0.749807145	97.2332820584229	66.0853240409511	138.012573921782	0.815879396
Mauritania	5.272733744	3.454128351	7.624888276	2.5033245	3.965316686	2.642023717	5.780381232	2.600929574	3.97926731071527	2.6534591405404	5.83715205075029	2.53582761	102.202737749607	66.7822461959066	149.288818529815	2.582678154
Mauritius	4.783767682	4.108046862	5.514993633	0.938173784	2.803110798	2.433709658	3.228812091	0.871783803	2.66721792412985	2.32240632098084	3.05047452907442	0.842756562	69.4120132203327	60.3100267128752	80.4647111118645	0.730098654
Mexico	1.981692657	1.728585429	2.303830415	1.006912709	1.302629164	1.141455326	1.492300585	0.944085108	1.32818705255547	1.16431002519432	1.51274957982695	0.902247169	31.305095410387	27.329414345715	35.6810195886369	0.876859207
Micronesia	5.9034161	4.041275851	8.407535963	-0.004097184	4.089241446	2.780319361	5.854703628	-0.083410418	4.19440492501796	2.85373858235842	5.98838996618151	-0.099873828	103.844518740913	70.2295847011532	149.347602089256	-0.060562295
Moldova	3.020618951	2.571153354	3.490889441	0.055857076	1.839892286	1.559465072	2.148819746	0.03214338	1.75371896072513	1.4863581663784	2.05794255246658	0.016347082	47.7101860683476	40.5123921621898	56.2707613278725	-0.033279409
Monaco	13.35977632	9.242514878	18.48083046	0.837151949	7.64605561	5.422943615	9.905276363	0.87811431	6.88609795172113	4.87275522826857	9.06528407599611	0.793404295	152.357406245886	108.453114735799	201.683055562535	0.668127731
Mongolia	21.95435713	18.36326239	27.99309238	0.432727917	15.48680217	12.78777904	19.48255118	0.351956095	16.426287957577	13.4840019786535	20.9079780977913	0.336968008	366.516602196771	302.858779337097	462.563298874883	0.37150776
Montenegro	3.500054739	2.649932538	4.585941349	0.572350713	2.175183426	1.646766068	2.838724783	0.65707533	2.1279323402	1.6157979859	2.775115641	0.695499329	55.256642464	41.98373621	73.08461407	0.494096364

									7085	3823	08271		2315	60808	01644	
Morocco	1.460334571	1.003252548	2.079301488	1.965225535	1.076090617	0.751991612	1.557094991	2.074220674	1.07867648272987	0.762887906982516	1.55311207016958	1.953229363	26.2276622315918	18.3168963581718	37.7198061797762	2.044412749
Mozambique	6.191419174	4.428168654	8.601286814	0.956047463	4.470284082	3.092041656	6.260127303	1.016263496	4.76242883442308	3.31467487968148	6.65485652170196	1.05332293	106.207209906484	73.2809882576748	149.255769769246	0.901953458
Myanmar	4.838588113	3.434193182	6.803195325	0.902902159	3.180916048	2.228104497	4.551564608	0.947826405	3.15169262199184	2.2309714511163	4.51376745554947	0.93012935	83.227395605699	57.8567106728238	119.196640254582	0.844820943
Namibia	2.803896357	1.9403833	4.03717449	0.781418206	1.763315409	1.266815097	2.455525237	0.849944753	1.70114240107684	1.23074511004426	2.36342093517592	0.859732122	50.8710169003629	35.916100975794	72.0098289288756	0.796524568
Nauru	5.655431198	3.820117658	7.97073779	0.496029589	4.08490256	2.807790519	5.936203055	0.570486208	4.09955055513117	2.82124324140107	5.95564914429168	0.564083974	110.032940171415	75.4783606455837	159.811779222156	0.524636841
Nepal	7.228915169	5.00466172	10.49900295	2.264311048	4.903385037	3.25064086	7.36580223	2.484119544	4.96679134648602	3.31898055805979	7.36952208454887	2.523613021	127.914655400459	84.2826887462575	191.422622392542	2.269149354
Netherlands	15.02340921	13.2655495	17.11450351	2.04444746	7.767074138	7.051649037	8.552801337	1.636876696	7.07924846192884	6.53018859996621	7.69858962733659	1.472717162	155.473241552251	144.409646896211	168.793008781099	1.200556858
Nevis	5.274716912	4.49358053	6.174535632	0.423658711	3.136151883	2.634809531	3.673935052	0.119931467	3.18334726311115	2.68396997926552	3.73625837002141	0.062357823	78.727175643443	65.5534250000256	92.2443900360885	0.196517565
New	9.8409	8.2470	11.901	1.621519	4.4740	3.9548	5.0457	1.020632	3.6978	3.2766	4.0893	0.768723	78.590	70.828	86.692	0.614733

Zealand	15475	25246	20706	221	87808	80627	38909	657	3626819582	4564519589	70484	57	262128891	1960470127	1448605157	053
Nicaragua	1.727619434	1.267308666	2.36962555	1.612760344	1.156476902	0.856717622	1.577876482	1.549766163	1.19305857886701	0.885064295568151	1.60971383245103	1.491222206	26.9698390901794	19.6712137507423	36.5296567222376	1.421192175
Niger	2.834852744	2.074151751	3.990719662	2.301599029	2.107562955	1.521056114	2.877891741	2.334634885	2.1720798719419	1.56840476033317	2.94311859928986	2.307933614	53.5356600966374	38.6398127462472	73.2848785949213	2.336366851
Nigeria	1.674828068	1.073201695	2.701347348	1.132493024	1.218756277	0.769655963	2.004798092	1.344685258	1.22633183958475	0.773723814838824	2.02349354141985	1.344063839	32.1555119712026	20.362977039033	52.3533700903203	1.299403573
Niue	2.81842988	1.826942283	4.359439114	-0.105605784	1.911106759	1.222349306	2.931327885	-0.075078498	1.93895858735388	1.25760554515789	2.92200181216848	-0.09998931	49.7998357397562	31.6631849515307	77.8119769434769	-0.067193052
North Korea	17.52713143	12.28539453	25.71724059	1.252926607	10.81012548	7.569051071	15.51333421	1.225544317	10.4534068997915	7.26975939230676	15.033036119793	1.185850048	257.420789029225	178.235160557086	368.734310888383	1.003923149
North Macedonia	2.028564774	1.579109705	2.602247099	0.604788201	1.310436516	1.035806172	1.648828298	0.653287461	1.31583284218244	1.02629058853041	1.64911630663118	0.684292255	33.1226031472067	26.0272334765038	41.6155924042481	0.485673831
Northern Mariana Islands	4.212332002	3.138434843	5.681290427	3.538269988	2.796313225	2.09982716	3.702815125	3.741724658	2.92872437700596	2.20820663381382	3.83049692849581	3.755595538	61.7560845410853	46.5246720335426	82.3091620393156	3.340222702
Norway	7.362316701	5.97234536	8.93918938	1.560780653	3.501639316	2.947699429	4.069213204	1.078149807	3.0332764862	2.5831462928	3.520748689	0.901915301	67.235902505	57.13113528	77.94511004	0.779252903

									8593	3667	53158		801	88327	11635	
Oman	2.303370263	1.624902467	3.183738203	2.216745536	1.504055296	1.066599467	2.055770262	1.562575212	1.5085743094974	1.07798081965172	2.0501242235238	1.368591613	32.4336792884542	23.4389884142614	44.5213691231716	1.409777633
Pakistan	12.16958741	9.109244402	16.7582626	1.694959104	8.015271497	5.978867091	11.01683474	1.725489078	7.99006943690238	5.96435812990186	10.9840847937926	1.683829045	214.97441697107	159.380415325244	295.571402308637	1.744966721
Palau	4.43854653	3.165683709	6.260503766	1.287393497	3.200487252	2.335667056	4.355154728	1.30177709	3.41939984685278	2.48992111049667	4.61093940176938	1.274143306	75.5135984347223	54.484158533487	102.653963613431	1.244932588
Palestine	1.49913031	1.11529341	2.044108589	1.9363157	0.945312161	0.69999049	1.265058982	1.560314401	0.951134998987491	0.692119427483828	1.26855141898846	1.41686333	21.0504509006821	15.514962588 4603	28.179001594 6842	1.412806071
Panama	1.898223146	1.619382446	2.24601972	1.303178902	1.189995	1.025364585	1.383986395	1.202819173	1.20605302420044	1.03597134017817	1.39782516721444	1.185098157	26.5424423270289	22.7542640775494	30.6716571467248	1.018996262
Papua New Guinea	3.802357404	2.478665099	5.736818097	2.043441814	2.640904225	1.702872244	3.988465617	1.998458837	2.74542219620683	1.81120215326659	4.10648360225451	1.951854862	65.2777344356879	41.650776739267	99.687066922 0498	1.982549774
Paraguay	7.048849605	5.919066816	8.190847635	2.495937393	4.477121752	3.739476317	5.306975772	2.295272935	4.57397855550783	3.83246368937986	5.41789210800446	2.250768689	111.184553150877	92.460647155 9432	131.245753861648	2.255975304
Peru	1.343214074	1.111187121	1.648425583	0.844397852	1.317805394	1.118130434	1.623504789	1.673328903	1.3883606360931	1.17465396287354	1.72592864468221	1.683864127	29.1811953533935	24.693248835 6599	35.857643333 5283	1.378341823
Philippin	1.8633	1.4642	2.3496	1.413265	1.2031	0.9424	1.5199	1.505586	1.1964	0.9362	1.5019	1.513148	31.048	24.529	39.215	1.354903

es	95647	10166	15876	711	07631	14926	52421	592	2632629476	35437730904	5735408627	291	3323723553	585734897	1212494791	023
Poland	4.437707223	4.043123072	4.81927611	0.238143064	2.862541543	2.619569518	3.112422438	0.23691674	2.8845513117668	2.64812643877944	3.13584511230488	0.253870522	74.1857678373903	67.7271363711079	80.4749115028752	0.141651058
Portugal	9.00079353	7.473182076	10.3968892	0.603322057	3.928728054	3.365212958	4.466046147	0.271199583	3.27427106931833	2.84579084128027	3.69073665707872	0.150412314	86.4425473350805	75.0154487280925	97.7781154197208	0.100660466
Puerto Rico	4.369370752	3.747599731	5.00896756	-0.20124442	2.287481488	2.00657759	2.589621822	-0.271887304	2.13705946811683	1.87154380365892	2.41746660326066	-0.285410341	53.679764473626	47.302823544559	60.6072347618506	-0.369784329
Qatar	1.904623785	1.327231253	3.075502264	2.462225613	1.131096953	0.80156245	1.677577961	1.498916955	1.10327288277739	0.780093494395895	1.60494349982535	1.109060189	21.1290359977371	14.8190486748597	31.6396861718706	1.161924029
Republic of Congo	10.27585462	6.892874825	14.92101519	1.575546098	7.995641596	5.429818129	11.10653722	2.14900185	8.25579374968299	5.59468422345273	11.3871841144799	2.177961781	202.55590966212	133.994806861975	285.270047680134	2.035081065
Romania	4.159220763	3.673084811	4.671094109	0.93226784	2.522600175	2.221640265	2.799138769	0.923124673	2.43418475282423	2.15118622537082	2.69001784404201	0.922401358	68.7559738140852	60.3469030011764	76.4520951536521	0.801698644
Russia	5.254301693	4.605155144	5.949532484	-0.125601512	3.107904691	2.746945577	3.491425738	-0.187139524	2.95926355803939	2.60839939490003	3.31698123824499	-0.210513074	79.7926870620121	70.3184999203839	89.7885393036856	-0.235980356
Rwanda	25.22286338	17.2173097	35.86616747	1.468613667	17.01151731	11.54567274	24.78816488	1.697869728	16.8818531379342	11.3756570479197	24.6550194645151	1.758376142	465.433044731556	312.732802699948	673.21260942502	1.448979071

Saint Kitts	5.274716912	4.49358053	6.174535632	0.423658711	3.136151883	2.634809531	3.673935052	0.119931467	3.18334726311115	2.68396997926552	3.73625837002141	0.062357823	78.727175643443	65.55342500 00256	92.2443900360885	0.196517565
Saint Lucia	5.989873945	4.996690777	7.02771262	1.133690834	3.670078002	3.020424919	4.407649106	0.968987835	3.61369435739822	2.99394794019879	4.34670261060964	0.906590564	95.4968319164371	78.5947414636353	115.173550916726	0.99051805
Saint Vincent	3.887829119	3.19902108	4.622555777	1.138770014	2.431743153	2.005492875	2.945106401	1.027508109	2.38392199935824	1.97289354251639	2.88017866265206	0.963506487	65.4220345224292	53.6703086962742	79.7597310091438	1.103293061
Samoa	1.413122749	0.948755464	2.02638263	1.292145032	0.947570079	0.622429322	1.344290543	1.484931498	0.936596934539489	0.627990777189362	1.32272383099332	1.441796819	24.2427994667922	15.8785875087339	34.2353904569397	1.421643168
San Marino	2.357209687	1.616374892	3.358624103	0.748957652	1.065402724	0.716565353	1.508049483	0.535828237	0.889417228757455	0.59965427574215	1.23475511472582	0.453946778	21.053873250911	14.6006799450881	30.2849120011869	0.316139185
Sao Tome and Principe	4.170994388	2.885196471	6.034849245	1.725519953	2.92578411	1.965687606	4.316548783	1.675792347	2.89473412261435	1.9324448200112	4.25590285124978	1.564666574	77.7738528122225	52.2944229613196	115.827057748863	1.821734283
Saudi Arabia	3.376002237	2.541741146	4.545152866	1.321246866	2.640745998	1.988809582	3.462183435	0.965817167	2.77607471749109	2.11722987468894	3.61391654113928	0.79941165	52.1225114240524	39.0565743421573	69.1830856230289	0.797597487
Senegal	3.887443252	2.658350136	5.623947444	2.455968324	2.606915857	1.772687031	3.698439841	2.614695184	2.61506136483702	1.79738123617786	3.69559640616798	2.615077179	69.2165497534151	46.6971093786429	98.7422491726556	2.517632767
Serbia	3.5767	3.0210	4.2910	0.110807	2.2014	1.8652	2.6217	0.120335	2.1454	1.8238	2.5435	0.126546	54.212	45.807	65.205	-0.01595

	58551	95808	19208	863	63374	28955	36657	696	3927534539	8003529412	7650088977	356	0525596351	1555759263	9327296286	6992
Seychelles	9.45575457	7.141377626	11.696066	1.760915019	5.561167214	4.229962926	7.004210813	1.502642576	5.30925047195394	4.03657316783919	6.58728705982911	1.389691308	144.462819831961	109.074945837971	182.681012683504	1.47352192
Sierra Leone	6.949258478	4.996790646	9.244870361	1.126803713	4.674377152	3.433878971	6.203575098	1.145149276	4.67339893005439	3.40297397589995	6.20337511716647	1.090028012	125.915166924459	92.5777040593741	166.508935335463	1.22051099
Singapore	9.27937987	7.492735773	11.31540756	2.71737731	3.061653099	2.574454982	3.551759837	1.261201083	2.0274208313428	1.74828317173358	2.28642187511279	0.667853058	45.0819367945579	39.1638573516797	50.9365385525238	0.477579835
Slovakia	5.519915288	4.932121139	6.244317076	0.293995162	3.376466478	3.019119294	3.777735764	0.304658658	3.28745563462166	2.95022014546067	3.66094758407984	0.31966293	88.4933771316654	77.8323689952752	99.7428318039146	0.166144751
Slovenia	5.263455978	4.3896564	6.177689322	0.406524277	2.654704546	2.319547968	3.042189499	0.217680601	2.33691418967238	2.05018419632393	2.67762294978653	0.141901671	58.8519801916385	51.2935009024317	67.4358292270184	-0.05415188
Solomon Islands	6.227375763	3.942115424	9.654642402	2.33136743	4.454859356	2.745575295	6.91460957	2.225517532	4.54000719153436	2.82488953844927	7.00766463677068	2.084083082	114.746130847574	70.385644883681	178.600472679676	2.422407005
Somalia	10.46318673	7.32623799	14.81566532	2.012977757	6.653848992	4.76140483	9.179793801	2.549054168	6.73845411516971	4.83792910115966	9.29803710006362	2.629182128	181.747959563928	131.008550591619	251.769051391427	2.323584118
South Africa	16.34458281	14.1402984	19.48048605	0.734130006	9.839595901	8.405531438	11.63770303	0.638574042	9.96663078897129	8.59412207110311	11.8440557670664	0.648932111	254.140561948577	216.282664520165	304.237343128944	0.589173355

South Korea	10.63312061	8.400758515	13.28305448	1.694555341	3.576248751	3.063971752	4.3510545	0.691286433	2.37206778016083	2.07481114115723	2.79962041328619	0.254031018	50.9691706877396	45.043276490904	61.9737478410339	-0.098295223
South Sudan	27.57219244	19.50823036	38.77338616	0.764809041	18.53226103	13.24580417	26.13478296	0.78336429	18.4084934572043	13.226133605489	25.7391246632528	0.762987493	520.136206973464	370.825725213627	738.497979807082	0.808988786
Spain	6.131954579	5.306752939	6.968412587	0.338389259	2.834810695	2.591436949	3.083986692	0.1303003	2.40369526345224	2.22959853876575	2.57273202588761	0.04407549	58.7795487939619	54.3416586117358	63.4081435434756	-0.095057648
Sri Lanka	11.96820304	9.741332357	15.46768637	2.458003408	6.042425628	4.98396401	7.413585497	1.993108665	5.59758468265258	4.67022580821288	6.85405723439972	1.9012275	142.533280079803	116.890313423708	176.636852200593	1.548558347
Sudan	6.253893647	3.274122226	9.680194625	1.554381718	4.252911339	2.089967533	6.768087046	1.38855254	4.41799109767635	2.18683390541958	7.01964591568199	1.32910878	99.0574163197345	49.164607566202	158.063501937778	1.323354328
Suriname	2.574387955	1.886135974	3.424988006	1.416319581	1.593502781	1.146162739	2.187566579	1.231359424	1.61268906596066	1.16592732050701	2.19111900148408	1.227223449	41.1725568036278	29.4430437648794	57.3691252075947	1.182484652
Swaziland	11.73562624	7.861598223	17.30800763	-0.051883744	6.861892917	4.732639058	9.756946358	-0.083966745	6.89642031399211	4.68223124720947	9.78644062120973	-0.026550053	185.518763815905	131.902902484155	266.344070993416	-0.189825536
Sweden	6.171546848	5.337787174	7.164113833	0.674112515	3.194782444	2.828032995	3.569448315	0.574310752	2.88945546643131	2.60191354639341	3.18314680584202	0.534574472	63.1130273680531	56.4384766814779	69.2588605937446	0.393917207
Switzerland	7.554276295	6.48512357	8.872870114	0.274803516	3.547302129	3.130983829	4.018326293	0.11079105	3.0579499550	2.6949270690	3.419225859	0.047939146	66.658945906	59.70345200	73.63006794	-0.134141028

									6576	9648	72811		3477	45968	12324	
Syria	1.144794177	0.838310342	1.553224387	1.71666554	0.78547673	0.576948017	1.048793672	1.618792569	0.829999008460301	0.613788400135103	1.1117157602431	1.596281177	16.8542606409795	12.432087097389	22.7527666341239	1.327541068
Taiwan	22.74783698	18.77801416	26.55727965	2.599567139	9.989701769	8.406039452	11.18196802	1.903740076	8.02686537480993	6.87968262070889	9.0068211447851	1.561479078	235.404073562368	201.559501375122	264.360644447791	1.542638972
Tajikistan	6.772924428	5.26377429	11.68844802	-0.215753132	4.980410811	3.861497316	8.580513968	-0.198182381	5.24639893186656	4.05617577310841	8.97580202977828	-0.199505739	117.635588700054	91.2738476163169	205.401251196918	-0.223271265
Tanzania	11.67271435	8.822389161	16.10359232	1.329064047	7.969636865	5.932185602	10.93799634	1.42285686	8.08784106563375	6.04612695307262	11.1921054073489	1.443565624	206.895353266187	153.234125942688	286.522265593962	1.346942645
Thailand	10.25545939	8.632359417	12.11830334	1.925669248	5.296119246	4.453096889	6.263648492	1.732866606	4.7295626691568	3.96865203424619	5.58382043179889	1.62285814	140.848527827832	117.501320574924	167.927945184283	1.510866565
Timor-Leste	4.388504839	3.092752088	6.458706331	1.767940306	2.710281569	1.915716739	3.911934246	1.890580021	2.66865593909442	1.88727473201854	3.83777791985368	1.931956776	69.9488806982826	49.0075542068723	101.095050177721	1.564881949
Tobago	3.647914029	3.085861842	4.227349848	1.020308329	2.317683365	1.988054789	2.714047847	0.91463239	2.35365224640255	2.00437149552807	2.74079186609421	0.888892159	57.964086754995	49.2915666118011	68.2319959226655	0.855663635
Togo	4.596086357	3.180702663	6.385353977	2.949654194	3.159420819	2.133094067	4.46700581	2.971667677	3.16850568055405	2.14982775274686	4.4944306889273	2.921400045	84.3435877220987	56.6336890206003	120.040837049486	2.999294657
Tokelau	1.9708	1.2863	3.1532	0.073551	1.4666	0.9522	2.3921	0.163610	1.5656	1.0232	2.5420	0.185115	35.465	22.717	57.671	0.142374

	21677	19768	40457	71	39032	77869	9484	994	5305706137	1987963875	4181419318	018	6773381201	4703749325	390810109	415
Tonga	3.178468036	2.180624646	4.618876906	0.421602148	2.292367404	1.564577243	3.351250988	0.505561989	2.34735956664347	1.61530267216376	3.41161575555112	0.491573483	55.2493875996994	37.176126273741	80.8989428008179	0.453770797
Trinidad	3.647914029	3.085861842	4.227349848	1.020308329	2.317683365	1.988054789	2.714047847	0.91463239	2.35365224640255	2.00437149552807	2.74079186609421	0.888892159	57.964086754995	49.2915666118011	68.2319959226655	0.855663635
Tunisia	2.387767855	1.787253063	3.261725508	2.796428054	1.187227049	0.879064735	1.57191018	2.089455383	1.14452439794235	0.846065137083243	1.49872669986608	1.941544139	26.4447185757961	19.7231665030082	34.5926907536984	1.841544305
Turkmenistan	12.86673539	11.68795055	14.2190676	-0.336699896	8.6005634	7.627398723	9.590984191	-0.348315657	1.58264980274958	1.19671519821908	2.05861672167247	-0.353172379	219.103299235678	194.804678964468	244.471999178065	-0.348661695
Tuvalu	5.391693125	3.513167509	8.026798043	0.562457124	3.838792297	2.514184857	5.939103944	0.538227476	8.72474216900901	7.77002923349658	9.72231510135 84	0.51779252	97.8963454562267	63.9780650385624	152.259667051658	0.524446302
Turkiye	2.808136039	2.244993477	3.678956306	1.159454697	1.637844043	1.261844872	2.138009281	1.022506444	3.92992087237078	2.59375564393933	6.0377870999511	0.971145639	36.441452795272	28.0781800229907	47.4974437998957	0.667762876
Uganda	22.8382653	17.07498159	30.94763593	1.344230476	13.95629969	10.73583637	18.82959933	1.95708787	13.7529855744277	10.5237280918066	18.5965138818843	1.859015916	385.545233569856	295.875130583844	526.072748906569	2.081030396
UK	14.36873567	12.71683767	16.08982792	0.758576337	8.104176138	7.377897588	8.726560915	0.63755428	7.63239017151452	7.03947795839113	8.16154549055824	0.613694994	164.919937088915	152.786533535652	176.07591286758	0.460597375

Ukraine	4.566315246	4.088179369	5.116393183	0.048879594	2.428043419	2.159794235	2.717423284	-0.020474226	2.15181790612277	1.91689547341629	2.40691435108924	-0.064490872	64.7175226110672	57.6128745168236	72.1945574742337	-0.048476825
United Arab Emirates	0.989892547	0.666687747	1.379178291	2.344040292	0.819505834	0.562628048	1.157068353	2.018699663	0.916526100327813	0.622270078719488	1.28799888964024	1.897145754	15.9439939530068	10.8442982312727	22.2819260942073	1.853656201
Uruguay	9.215369636	8.102776061	10.3008248	-0.06550463	5.670145393	4.975931531	6.252306011	-0.108498309	5.56989305490681	4.90725836356424	6.12743629383234	-0.118795193	129.558500060639	113.696772699089	142.298667158512	-0.176736803
USA	8.875688164	7.64789388	10.19005297	0.908917135	4.146824743	3.667920367	4.55427149	0.821481857	3.51020542514761	3.19239274666286	3.83122557441056	0.794144438	84.0246099899231	76.5600608961426	91.2501054124583	0.648181702
Uzbekistan	6.415247529	6.058669963	6.814257971	-0.27971447	4.261217403	4.052266091	4.520914476	-0.305704873	4.35925461462091	4.14632033282941	4.63893528372854	-0.312084977	104.348901565714	99.3318643570217	110.474066388618	-0.324301494
Vanuatu	2.872148611	1.92186828	4.255521462	1.726337285	1.866022746	1.269247585	2.732507634	1.715165504	1.8782543294564	1.28485133854667	2.74365633376915	1.673331483	51.0130576220027	34.1299612190382	76.0153636471952	1.754572553
Venezuela	3.085964706	2.813672628	3.397241612	1.207900874	2.096031231	1.896490738	2.33918345	1.194910482	2.20054956895831	1.99013007842889	2.4427020452691	1.185810814	49.4381545065249	44.6995641986195	55.4840614715385	1.108391172
Vietnam	4.940286368	3.287976676	7.376283268	3.851338529	2.844235875	1.692588685	4.456043439	3.257997408	2.63957251357092	1.54584620829429	4.0858249929487	2.960714362	74.5024735024806	44.733492042264	116.422293212637	3.283833688
Virgin Islands	3.43115772	2.813286438	4.072052657	0.11076913	2.047593772	1.736966842	2.379617069	0.211755334	1.9567262219	1.6607236476	2.261211783	0.244506927	52.028420923	43.73751735	60.76226501	0.046253133

									9645	6749	76822		621	46544	75898	
Yemen	4.500592529	2.102623941	6.645897324	1.690651899	3.197543035	1.506673321	4.737653537	1.538424168	3.40183978094796	1.60059596520359	5.01580992451924	1.516527389	73.9806963565399	34.9749261983806	108.223476238177	1.50936323
Zambia	12.3858758	8.926888162	17.00854678	1.79386712	8.80320588	6.2255116	11.83763186	2.245575164	8.6852211916879	6.24516400606414	11.6633867976063	2.17326606	245.792361696993	172.038728791193	335.471587015833	2.335778544
Zimbabwe	13.54263771	9.559089864	19.75564257	0.583471638	8.665328281	6.212828199	11.67509341	0.756526327	8.62979278436592	6.225081793	11.5178964834792	0.728622393	236.143049738998	165.233859060638	322.93420274809	0.842047592
